# Effect of Breastfeeding Promotion on Early Childhood Caries and Breastfeeding Duration among 5 Year Old Children in Eastern Uganda: A Cluster Randomized Trial

**DOI:** 10.1371/journal.pone.0125352

**Published:** 2015-05-04

**Authors:** Nancy Birungi, Lars T. Fadnes, Isaac Okullo, Arabat Kasangaki, Victoria Nankabirwa, Grace Ndeezi, James K. Tumwine, Thorkild Tylleskär, Stein Atle Lie, Anne Nordrehaug Åstrøm

**Affiliations:** 1 Department of Global Public Health and Primary Care, Centre for International Health, University of Bergen, Bergen, Norway; 2 Department of Clinical Dentistry, University of Bergen, Bergen, Norway; 3 Department of Dentistry, School of Health Sciences, College of Health Sciences, Makerere University, Kampala, Uganda; 4 Department of Epidemiology and Biostatistics, School of Public Health, College of Health Sciences, Makerere University, Kampala, Uganda; 5 Department of Epidemiology, Mailman School of Public Health, Columbia University, New York, United States of America; 6 Department of Paediatrics and Child health, School of Medicine, College of Health Sciences, Makerere University, Kampala, Uganda; The National Institute for Health Innovation, NEW ZEALAND

## Abstract

**Background:**

Although several studies have shown short term health benefits of exclusive breastfeeding (EBF), its long term consequences have not been studied extensively in low-income contexts. This study assessed the impact of an EBF promotion initiative for 6 months on early childhood caries (ECC) and breastfeeding duration in children aged 5 years in Mbale, Eastern Uganda.

**Methods:**

Participants were recruited from the Ugandan site of the PROMISE- EBF cluster randomised trial (ClinicalTrials.gov no: NCT00397150). A total of 765 pregnant women from 24 clusters were included in the ratio 1:1 to receive peer counselled promotion of EBF as the intervention or standard of care. At the 5 year follow-up, ECC was recorded under field conditions using the World Health Organization’s decayed missing filled tooth (dmft) index. Adjusted negative binomial and linear regression were used in the analysis.

**Results:**

Mean breastfeeding duration in the intervention and control groups (n=417) were 21.8 (CI 20.7–22.9) and 21.3(CI 20.7–21.9) months, respectively. The mean dmft was 1.5 (standard deviation [SD] 2.9) and 1.7 (SD 2.9) in the intervention and control groups, respectively. Corresponding prevalence estimates of ECC were 38% and 41%. Negative binomial regression analysis adjusted for cluster effects and loss-to-follow-up by inverse probability weights (IPW) showed an incidence-rate ratio (IRR) of 0.91 (95% CI 0.65–1.2). Comparing the effect of the trial arm on breastfeeding duration showed a difference in months of 0.48 (-0.72 to 1.7).

**Conclusion:**

PROMISE EBF trial did not impact on early childhood caries or breastfeeding duration at 5 years of age. This study contributes to the body of evidence that promotion of exclusive breastfeeding does not raise oral health concerns. However, the high burden of caries calls for efforts to improve the oral health condition in this setting.

**Trial Registration:**

ClinicalTrials.gov NCT00397150

## Introduction

Exclusive breastfeeding (EBF), that is giving the baby no solids or liquids besides breast milk, other than vitamins and medication has been considered to be one of the most effective preventive strategies to reduce infant mortality in developed and low income countries [1–5]. With respect to optimal duration of exclusive breastfeeding, a Cochrane review concluded that EBF for six months has advantages over EBF for three to four months such as reduced risk of gastrointestinal infection and more rapid maternal weight-loss after birth [6]. Another Cochrane review concluded that additional lay support for breastfeeding mothers was effective in prolonging EBF, whereas the effect on duration of any breastfeeding was uncertain [7]. A review focusing on breastfeeding promotion by peer counsellors and summarizing the small amount of evidence from Africa, revealed improvements in terms of breastfeeding initiation, duration and exclusivity [8]. Although several studies have shown some short term health benefits of exclusive breastfeeding promotion, its long-term consequences have rarely been studied in low-income contexts. Little is known about the long term effects of exclusive breastfeeding promotion on early childhood caries (ECC) and duration of any breastfeeding, particularly in non-occidental cultural settings [9,10].

Early childhood caries denotes any form of caries (cavitated or not) occurring in the primary dentition of children 71 months or younger [11,12]. ECC is one of the most prevalent chronic childhood diseases having extensive quality of life implications for the child as well as the child’s family [13–16]. Previous studies from Uganda have reported a caries prevalence of 18% in 6–36 months old children [17]. A study conducted in Kampala, the capital city of Uganda, involving preschool children aged 3, 4 and 5 years revealed caries prevalence of respectively, 45%, 59% and 65% indicating that ECC is a significant problem among preschool children in this country [18]. There is conflicting evidence as to how breastfeeding impacts on ECC with some studies reporting a positive, some a negative- and others no relationship between breastfeeding and ECC [19–21]. Iida et al.[22] concluded that infant breastfeeding and its duration whether overall, full or exclusive was not associated with any increased risk of ECC. In Japanese 3- year- old children, however, breastfeeding for 18 months or longer was associated with increased prevalence of dental caries [23]. Prolonged duration of breastfeeding above 1 year and nocturnal breastfeeding has been associated with ECC development [24]. Evidence suggests that teeth are susceptible to caries shortly after tooth eruption and prior to final maturation which may indicate that EBF practices during the first six months could be important for oral health—potentially as a risk factor [25]. Evidence of a beneficial or harmful effect of breastfeeding on ECC has been provided mainly by observational studies and thus could be attributed to methodological limitations [26]. According to systematic reviews on the relationship between breastfeeding and dental caries in children, only three studies had moderately appropriate design, but were without uniform definition of EBF [10,27]. However, the Belarussian EBF promotion intervention designed as a cluster randomised trial to promote exclusive- and prolonged breastfeeding showed no significant effect on ECC at 6.5 years follow-up [28]. The feeding patterns in this Belarussian population of mothers are quite different from what is commonly practiced in several low-income countries in Sub-Saharan Africa, including Uganda [29]. Assigning some participants to a breastfeeding arm and others to a non- breastfeeding arm would be unethical, so a trial studying the effect of promotion of EBF provides a good opportunity to study the effect of this intervention on ECC.

Assuming that EBF promotion may change the general health and oral health focus of the caretakers, it could impact on children’s subsequent health and oral health, including duration of breastfeeding and feeding patterns as well as early childhood caries. Thus, this study assessed the effect of promoting EBF for 6 months on ECC and breastfeeding duration assessed at 5 year follow-up in children enrolled at birth to the PROMISE EBF trial in Uganda.

## Subjects and Methods

The protocol for this trial and supporting CONSORT checklist are available as supporting information; see S1 Checklist and S1 Protocol.

### Study setting

The present study is a five year follow- up of caretaker-children pairs of the Ugandan site of the PROMISE-EBF trial (ClinicalTrials.gov no: NCT00397150) conducted in 2011in Mbale district, Eastern Uganda [30]. This district has a literacy rate of 75% and 60% among males and females, respectively [31]. The fluoride concentration in drinking water is not monitored and may vary across the different geographical regions.

### Study design

PROMISE-EBF was a multicentre community based cluster-randomised behavioural intervention trial conducted in sub Saharan Africa between January 2006 and June 2008. The aim of this intervention was to assess the effect of individual home-based peer counselling to promote exclusive breastfeeding for 6 months after birth. The unit of randomization were clusters made up of 1–2 villages with an average of 1000 inhabitants corresponding to a birth rate of approximately 35 per cluster. A total of 24 clusters were stratified into urban and rural and allocated at random (computer generated with an allocation ration 1:1) to intervention and control groups. Women in the intervention group received home based individual peer counselling to support EBF for 6 months from lay counsellors in terms of information and encouragement in 5 visits. One visit was prenatal and the other visits were in the first, fourth, seventh and tenth week post-delivery. The control group received standard care from the public health services. The primary outcome of this trial was prevalence of EBF and diarrhoea reported by mothers of infants aged 12- and 24 weeks. Detailed information about PROMISE-EBF has been published previously [5,32].

**Fig 1:**
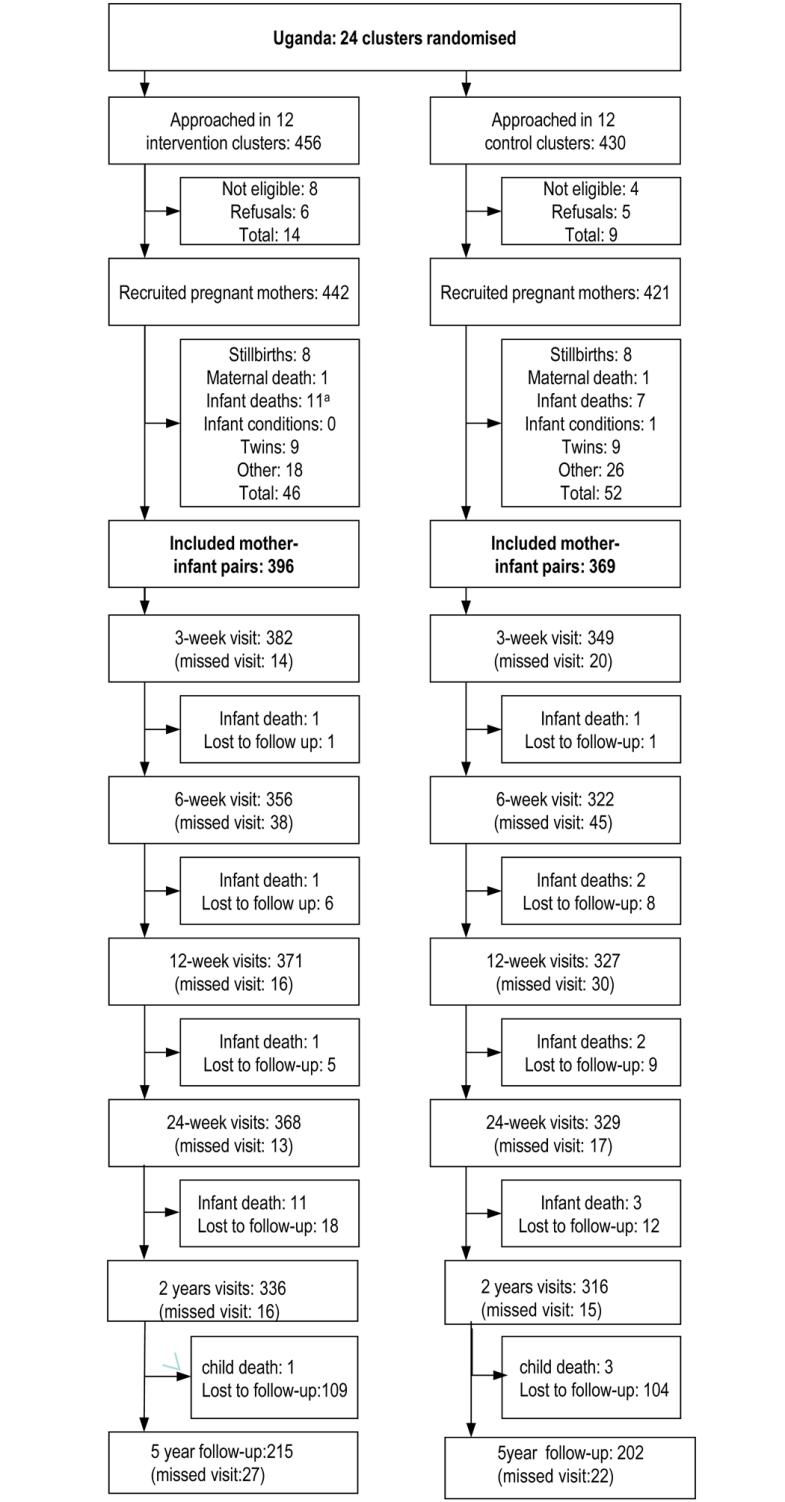
Flowchart. ^a^Some had more than one inclusion criteria.

The PROMISE-EBF study involved 765 healthy mother-infant breastfeeding pairs and resulted in two child cohorts from the intervention and control groups that differed substantially with respect to the prevalence of EBF at 24 weeks of infant’s age. (59% versus 12%) [5]. The visits and follow-ups were carried out at household level in the 24 clusters between 2006–2011 including in the intervention and control groups respectively; 336 and 316 mother-child pairs from the 2-year follow-up and, 215 and 202 mother-child pairs in the 5-year follow-up (Fig 1). A proportion was lost-to-follow-up due to relocation or not being at home when approached for two to three home visits.

### Primary and secondary outcomes

The primary outcome of this study is the prevalence of ECC assessed by the decayed missing filled teeth (dmft) index at the five year follow-up.

The secondary outcome of this study is the duration of any breastfeeding.

### Interview with mothers at the 5-year follow-up visit

Research assistants conducted structured interviews with mothers/ caretakers in their local language Lumasaba. The interview included questions regarding socio-demographic characteristics, general health, breastfeeding, nutrition, food security, morbidity, oral health and oral health related quality of life (OHRQoL). Breastfeeding duration was assessed by mothers recall at 2- and 5 years follow-up. The questions; did you breastfeed, are you still breastfeeding and for how long did you breastfeed were used to evaluate breastfeeding duration at all interview visits. Multiple correspondence analyses was used to construct a socio-economic index categorised into wealth quintiles and based on ownership of assets such as furniture and household characteristics including electricity, type of water source, roof material and toilet type from the recruitment interview. Multiple correspondence analysis is analogous to principal component analysis for categorical data [33].

### Clinical oral examination of children at the 5-year follow-up visit

A full mouth clinical oral examination was carried out at household level by two trained and calibrated dentists (NB and AK). Children were examined following the WHO guidelines under field conditions [34]. Children were placed with their face in upward direction facing natural light, with the clinician standing or sitting at the backside using a mirror and probe for oral examination. ECC was assessed on fully erupted teeth using the decayed, missing filled teeth index (dmft) [34]. A tooth was recorded as decayed if it was visually cavitated or if on probing, the probe stuck into the suspected tooth surface. A missing tooth was qualified as missing due to extraction when this was confirmed by the caretaker. Further confirmation was sought if caries was the reason for extraction. The primary outcome variable; ECC, was constructed from the dmft index. In the present analysis ECC was used as a count variable and also dichotomised. The count variable was a sum score of decayed, missed and filled teeth in child’s mouth. The count variable was dichotomised into: dmft>0 denoted presence or prevalence of ECC and dmft = 0 denoted absence of ECC. The interviewers and dentists were aware of the children’s involvement in the PROMISE-EBF trial but were blinded with respect to their group allocation. Duplicate oral examinations were carried out by dental surgeons (NB and AK), involving 22 children considered to be representative of the trial participants based on age and site of residence.

### Reliability measurement

Un-weighted Cohen’s Kappa was used to assess inter-rater reliability by comparing the dmft score for each tooth across two examiners. Intra-rater reliability was assessed by comparing the dmft score for each examiner across a time interval of two weeks. The observed median Kappa for inter-rater agreement amounted to 0.92 with an interquartile range (IQR) of (0.63–1). The intra-rater agreement revealed a median Kappa of 0.80 IQR (0.64–1).

### Data cleaning and statistical analysis

Double data entry was carried out. The statistical package Stata IC version 13 was used for data analysis. Analyses of the sample characteristics were performed using frequency tables, means and proportions. Due to overdispersed and skewed count data, with about half of the cases presenting with dmft = 0, the standard negative binomial regression was used. In a parallel analysis, a two-step zero inflated negative binomial regression model was employed in order to predict a zero or non-zero ECC outcome in the first step of analysis using logit link. In the second step, the effect of the intervention on the non-zero ECC outcomes was estimated. The goodness fit of standard negative binomial model was compared with the goodness of fit of the zero inflated one using the Vuong’s test. Incidence rate ratios (IRRs) and 95% confidence intervals (CIs) were used to assess the effect of breastfeeding promotion on ECC experience whilst adjusting for the cluster design. Linear regression for continuous normally distributed data was conducted to compare any breastfeeding duration between the trial arms. Breastfeeding recall at 2- and 5 years follow-up was assessed using survival analysis for time related data and described using a Kaplan Meier plot. To adjust for potential differences in loss-to-follow-up between the trial arms, an inverse-probability weights method was applied. A probit regression analysis was conducted to assess background factors which were associated with lost to follow-up (socio-economic status, level of education and residence in rural/urban area). This probability was then used to calculate the inverse probability weight by calculating the inverse of predicted scores for being lost to follow-up. These weights were included in the regression models (using the pweight command). The median of the weights was 1.8 (IQR 1.7–2.0); i.e. children who were available for oral examination were weighted slightly up in the analysis to represent a median 1.8 children at baseline.

### Ethics

Ethical approval for the study was granted by Makerere University Medical School Research Ethics Committee, the Uganda National Council for Science and Technology and Regional Committees for Medical and Health Research Ethics, Western Norway (05/8197). Consent was given at the individual level by participants after cluster randomization. As a first step, verbal consents were obtained from the pregnant women as to whether or not she wanted to be visited by a data collector for more information about the trial. In a second step, written consent was given after comprehensive information about the trial procedures up to the last follow-up. At the 5- year- follow-up, the caretakers gave assent for their children. Signed or thumb-printed informed consent was obtained from each mother prior to study participation. The consent procedure was approved by the ethical committees.

## Results

Of the 417 children examined, 208 were boys and 209 girls, with an almost balanced sex distribution across the trial arms. The median age was 4.5 (IQR 4.2–5.2) years. At baseline the intervention and control group differed to some extent with respect to socio-economic status (including electricity in the house, water source) and place of birth (Table 1). The socio-demographic differences at the five year follow-up paralleled those seen at baseline (randomization) with significant differences in the socio-economic status and place of birth categories (Table 2). Continuous data did not differ by allocation status at baseline and 5year follow-up. Loss to follow-up in the 5-year follow-up was slightly more likely among those who were primipara, single, widowed, separated or divorced (Table 2).

**Table 1:** Baseline characteristics at randomisation.

	Intervention	Control
**Categorical data**		
Eligible mother infant pairs(n)	396	369
Marital status		
Married	244(62%)	234(64%)
Cohabiting	119(30%)	104(28%)
Single, widowed, separated, or divorced	29(7%)	28(68%)
Socio-economic status quintile		
1(poorest)	91(23%)	62(17%)
2	97(24%)	86(23%)
3	76(19%)	49(13%)
4	71(18%)	84(23%)
5(least poor)	61(15%)	88(24%)
Electricity in house		
Yes	53(14%)	70(19%)
Toilet		
None or open	84(25%)	59(18%)
Pit or ventilated improved pit	245(72%)	266(81%)
Flush	10(3%)	3(<1%)
Parity		
Primipara	81(21%)	85 (23%)
Multipara	311(79%)	281 (77%)
Previous child death		
Yes	109(36%)	80(29%)
Attendance of antenatal (index child)		
Yes	272(72%)	274(78%)
Place of birth (index child)		
Out of facility	208(55%)	146(42%)
Facility	173(45%)	205(58%)
**Continuous Data Median (IQR)**		
Maternal Age		
Years	25(20–30)	24(20–30)
Maternal Education		
Years	6(4–8)	6(5–9)
Maternal Body Mass index		
At 6 weeks (kg/m^2^)	22(20–24)	22(20–24)

IQR- Interquartile range, kg\m^2^- kilogram per square metre.

**Table 2:** Background characteristics at 5 years follow-up and among that lost- to-follow-up.

	Intervention % (n)	Control % (n)	Lost to follow-up in intervention % (n)	Lost to follow-up in Control % (n)
**Categorical data**				
Eligible mother pairs	54.3(215)	54.7(202)	45.7(181)	45.3(167)
Marital status				
Married, cohabiting	92.5(197)	95.5(193)	92.7(166)	88.5(146)*
Single, widowed, separated or divorced	7.5(16)	4.5(9)	7.3(13)	11.5(19)
Social economic status quintile				
1 (poorest)	70.7(152)	58.6(119)*	61.9(112)	47.3(79)
2 (least poor)	29.3(63)	41.4(84)	38.1(69)	52.7(88)
Electricity in house				
Yes	12.7(27)	16.6(33)	14.6(26)	22.8(37)
Toilet				
None or open	25.4(46)	22.3(41)	24.0(38)	12.5(18)
Pit or ventilated improved pit/Flush	74.6 (135)	77.7 (143)	76.0 (120)	87.5(126)
Parity				
Primipara	16.6(35)	20.9(42)	27.8(50)*	28.8(46)
Multipara	83.4(176)	79.1(159)	72.2(130)	71.2(114)
Previous child death				
Yes	34.3(61)	32.7(52)	37.2(48)	23.7(28)
Attendance of antenatal (index child)				
Yes	70.4(145)	79.4(154)	74.7(127)	76.0(120)
Place of birth (index child)				
Out of facility	58.1(122)	39.9(77)*	50.3(86)	43.7(69)
Facility	41.9 (88)	60.1(116)	49.7(85)	56.3(89)
Child sex				
Male	50.2(108)	49.5(100)	51.4(92)	52.4(87)
Female	49.8(107)	50.5(102)	48.6(87)	47.6(79)
**Continuous data median (IQR)**				
Maternal Age				
Years	26(21–30)	25(25–31)	24(20–28)	23(20–28)
Maternal Education				
Years	6(4–7)	6(4–9)	6(4–8)	7(5–9)
Maternal Body Mass index				
At 6 weeks (kg/m^2^)	22(20–24)	22(20–24)	22(20–23)	23(20–23)
Child Age				
Years	4.6(4.2–5.2)	4.4(4.1–5.1)	4.5(4.1–5.0)	4.1(3.9–5.0)

*p<0.05 IQR—interquartile range, kg\m^2^- kilogram square metre.

The prevalence of children with caries in the intervention and control arm was 38% and 41%, respectively. The corresponding mean dmft was 1.5 (SD 2.9) and 1.7 (SD 2.9), respectively (Table 3). Mean dmft for boys was 1.6 in both trial arms, whereas for girls the mean dmft was 1.5 (2.7) and 1.8 (2.9) in intervention and control arm, respectively (Table 3).

**Table 3:** Mean early childhood caries in total dentition and anterior maxillary teeth, mean breastfeeding duration and proportion of early childhood caries at 5 years follow-up in intervention (n = 215) and control group (n = 202).

		Intervention		Control	
	n	Mean(SD)	Median (IQR)	Mean (SD)	Median(IQR)
ECC in all dentition					
Boys	208	1.6 (3.2)	0 (0–2)	1.6 (2.9)	0 (0–2)
Girls	209	1.5 (2.7)	0 (0–2)	1.8 (2.9)	0 (0–2)
Overall sample	417	1.5 (2.9)	0 (0–2)	1.7 (2.9)	0 (0–2)
ECC in anterior maxillary teeth					
Boys	208	0.54 (1.2)	0 (0–0)	0.34 (1.1)	0 (0–0)
Girls	209	0.51 (1.3)	0 (0–0)	0.43 (1.2)	0 (0–0)
Overall sample	417	0.53 (1.3)	0 (0–0)	0.38 (1.1)	0 (0–0)
**Mean breastfeeding duration in months**	**n**	**Mean (SD)**		**Mean (SD)**	
	417	21.8 (6.5)		21.3 (6.2)	
**Proportion of ECC > 0**		**% (n)**		**% (n)**	
		38 (81)		41 (83)	

ECC-Early childhood caries, SD-standard deviation, n-number, IQR- Interquartile range.

**Fig 2:**
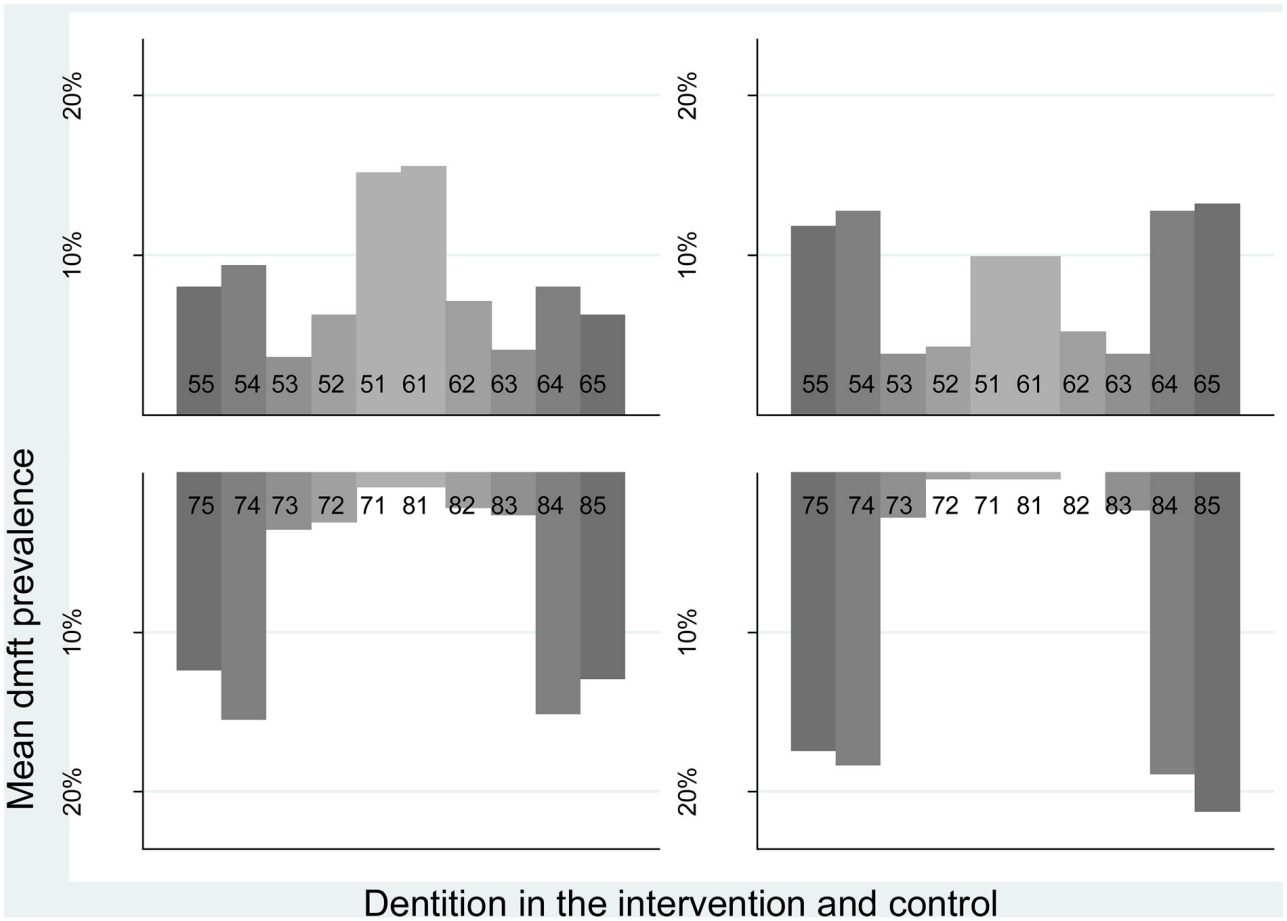
Mean caries (dmft) prevalence distribution by tooth type (numbered) in maxilla (upper figures) and mandible (lower figures) in intervention (left figures) and control group (right figures).

As shown in Fig 2, the tooth specific pattern of ECC was similar across trial arms. Maxillary (upper jaw) central incisors and mandibular (lower jaw) molar teeth were most frequently affected by ECC in the intervention and the control arm. In the upper jaw, the mean dmft for the central incisors (teeth 51, 61) were highest in the intervention arm, whereas the mean dmft for the molar teeth (teeth 55, 54) were highest in the control group. In the lower jaw, the mean dmft for the molar teeth (teeth 74, 75) were highest in the control group.

**Fig 3:**
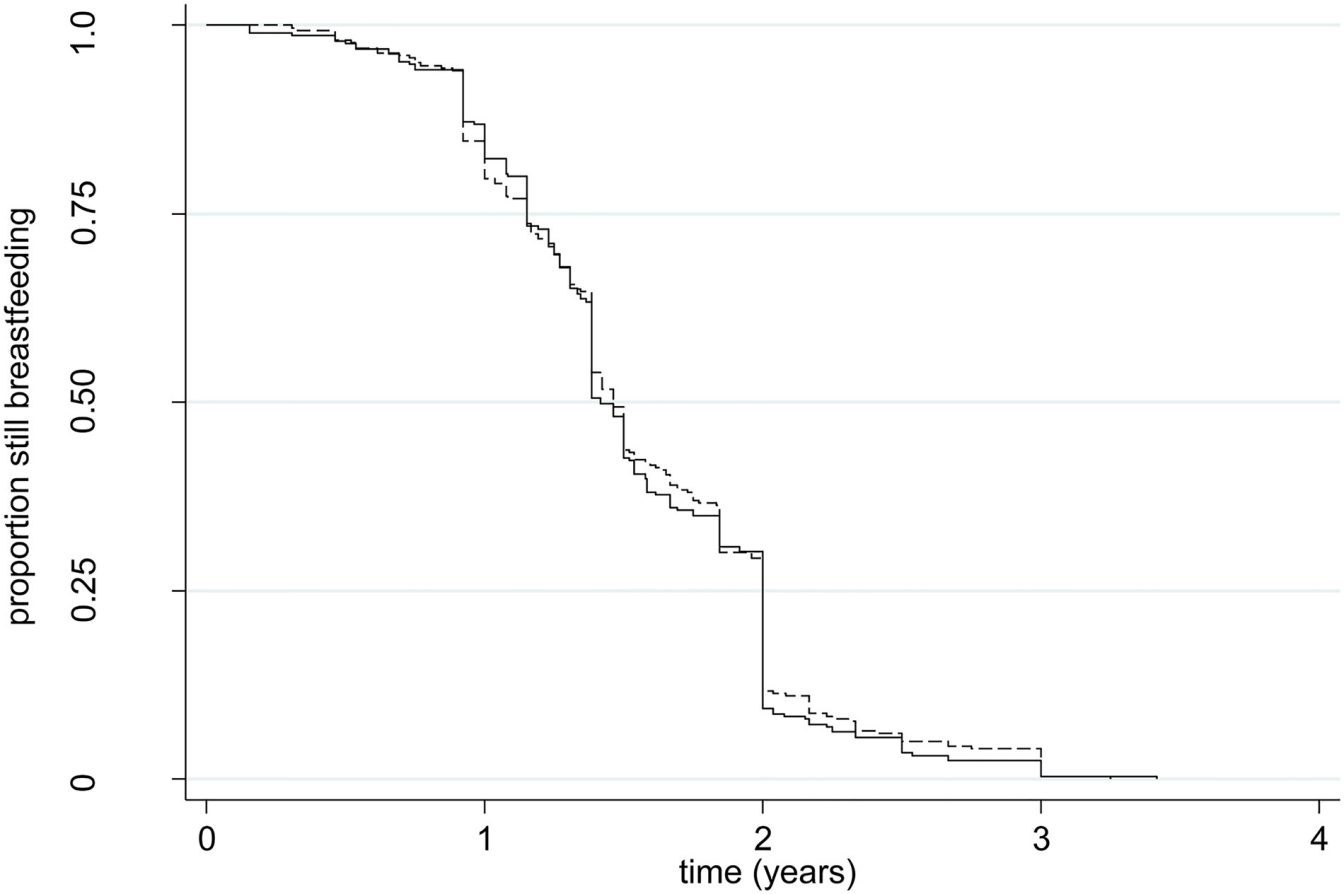
Kaplan-Meier plot showing breastfeeding duration in years. * Dashed line indicates intervention group; complete line indicates control group.

Mean breastfeeding duration was 21.8 (CI 20.7–22.9) months in the intervention and 21.3 (CI 20.7–21.9) months in the control arm (Table 3) (Fig 3).

Linear regression, adjusted for cluster effect revealed no statistically significant relationship between breastfeeding duration and the trial arms with a month’s difference of 0.48 (CI -0.72 to 1.7) (not shown in Table).

The negative binomial regression, with robust variance estimates adjusted for clustering, showed no significant difference between the trial arms with respect to total dmft and dmft in anterior maxillary teeth. Compared to the control arm, the incidence rate ratio of having ECC in the intervention arm was 0.91 (CI 0.65–1.26). The corresponding ratio for ECC in anterior maxillary teeth was 1.31 (CI 0.84–2.31). The estimates adjusted for site and socio-economic status were similar to the unadjusted ones with IRRs of 0.92 (C1 0.67–1.27) and 1.42 (0.88–2.31) respectively (Table 4). Zero inflated negative binomial analyses revealed essentially the same estimates as the negative binomial regression model (S1 Table).

**Table 4:** Incidence rate ratios (IRR) with 95% confidence intervals (CI) for early childhood caries (ECC) in all dentition and in anterior maxillary teeth in intervention and control groups (n = 417), both unadjusted (except for clustering) and adjusted negative binomial regression models.

	ECC in all dentition	ECC anterior maxillary teeth
	IRR(95%CI)	IRR(95%CI)
**Unadjusted**		
Intervention	0.91(0.65–1.23)	1.39(0.84–2.31)
Control	1	1
**Adjusted** *		
Intervention	0.92(0.67–1.27)	1.42(0.88–2.31)
Control	1	1

* adjusted for site of residence and socio-economic status in addition to clustering.

## Discussion

Although the PROMISE-EBF trial had a substantial impact on breastfeeding exclusivity, the intervention had no effect on breastfeeding duration as reported by mothers at the 2-and 5- year- follow-up visits. Thus, this study found no significant differences in ECC and breastfeeding duration, corresponding to the null hypothesis. However, several studies may not identify true differences (type II errors).

Whereas 59% and 12% of mothers in the intervention and control arm reported exclusive breastfeeding at 24 weeks follow- up, mean duration of any breastfeeding at 5 year follow- up was 21.8 and 21.3 months in the intervention and control groups respectively. These results are close to the WHO recommendations of complementary feeding for at least two years [1,2] and corroborate estimates of previous studies from East African countries with mean number of months having breastfed reported to be in the range from 13.0–19.6 months [4]. The lack of effect on duration of any breastfeeding might be related to the fact that the current cultural practice in Uganda, represented by the control arm, is not far from the recommendations in terms of total duration of breastfeeding [2].

Previous studies of observational design have reported a positive association between ECC and breastfeeding [35–37]. However, the present PROMISE-EBF trial using home-based exclusive breastfeeding promotion by peer counsellors provided to mothers during the first weeks after birth, did not show any impact on ECC across early childhood. The present results are in line with a similarly designed study in a different setting in Belarus [28]. They are also accordant with another western study focusing caries reduction as an outcome of peer led social support for recommended breastfeeding practices [38]. Although the large Belarusian trial led to potentially clinically important increase in exclusive and total breastfeeding duration, the trial was found to be without any effects on ECC in children at the 6 5-years follow-up visit. It is evident that intervention strategies, such as PROMISE EBF, have been more successful in modifying behavioural and attitudinal outcomes such as parents’ cognition and feeding practices and less successful in reducing incidence of dental caries [28,39]. In contrast to the present results, a recently conducted Brazilian study reported that early home based dietary counselling during infancy, including exclusive breastfeeding for 6 months, actually reduced caries incidence and severity at 4 years of age in this low-income setting [40]. Notably, the specific effect of exclusive breastfeeding promotion in that study was difficult to assess as feeding practices was only a part of the intervention packages.

Despite the absence of any intervention effect in the present study, substantial proportions of the participating children presented with ECC at 5 years of age, amounting to 38% and 41% in the intervention and control arm, respectively. These rates are consistent with previously recorded estimates among preschool children in Uganda [18]. Carious lesions were not evenly distributed across teeth, being most prevalent in the upper incisors and the lower molars and less prevalent in the lower incisors, thus reflecting the pattern of eruption. Consistent caries patterns have been reported previously in a similar population and socio-cultural setting [17,18]. Information about the prevalence of ECC in the paediatric population of sub-Saharan Africa is scarce and the Mbale region, eastern Uganda has been surveyed to a very limited extent. The results regarding ECC prevalence and distribution reflect an unmet need for dental care, suggesting that ECC constitutes a public health problem in the area investigated. A high prevalence of ECC as observed in the present study of Ugandan preschool children, may be attributed to low levels of fluoride in drinking water, lack of wide spread use of fluoridated toothpaste and to the ongoing nutrition transition in the region. The nutrition transition implies risk factors for health and oral health such as the adoption of diet high in fat and commercialised sugar products. [41]

A number of strengths and limitations of the present study merit consideration. An important strength was the randomized trial design utilised that minimised the risk of confounding. However, the PROMISE-EBF intervention was designed to assess changes in exclusive breastfeeding during the first 6 months and not to compare duration of breastfeeding or ECC at 5 year follow- up. The extent of losses to follow-up, typical of long-term trials in resource poor settings, reduced the analytical sample for its ability to differentiate smaller intervention effects. Nevertheless, post-hoc power calculations showed that the power of this study was satisfactory in terms of assessing duration of breastfeeding and ECC. The substantial loss-to-follow-up was largely due to relocation of the families to unknown addresses. This high mobility of the studied population could have contributed to potential group differences in the intervention and control arms and potentially to a selection bias. Checking baseline characteristics of socio-economic-and educational factors between those followed-up and those lost-to-follow-up at 5 years suggests that there is a slightly higher loss of those most educated and of the socio-economically least poor. Thus, compared to individuals retained in the cohort, individuals lost to follow-up tended to be advantaged in terms of socio-economic status. However, the loss to follow—up was rather balanced between the trial arms, making it less probable that bias due to non-response has seriously affected the present effect estimates. Adjusting for loss-to-follow-up with inverse probability sample weights, IPW, taking socio-economy, years of education of the mother and site of residence into account did not change the results (only second decimal changes). It is thus not likely that loss-to-follow-up biased the findings substantially.

To protect against measurement bias interviewers and dentists at the 5 year follow-up were blinded to the allocation status of the participants and had not been involved in the previous follow-ups. However, without x-ray examination, enamel caries or white spot lesions may have been overlooked or misclassified leading to an underestimation of ECC prevalence. To limit such misclassification, the dental recorders were trained on the relevant examination and they were calibrated. Both examiners were experienced dental surgeons, practicing clinical dental work at the time of the study. The acceptable levels of inter- and intra-rater reliability measures [42] obtained suggests that misclassification, being a threat to the internal validity of the clinical registration may not be a substantial problem in this study. On the other hand and since it was impossible to fully blind participants, social desirable responses may have been given and mothers in the intervention arm may have reported healthier practice just to please the research staff. If the tendency of social desirable answers with respect to breastfeeding duration differed across study groups, this may have constituted a source of differential misclassification providing a biased estimate of the observed association. However, the fact that the intervention group did not report a longer duration of breastfeeding than the control arm could suggest that this may not be a substantial problem. Some studies focusing on breastfeeding have shown that recall tends to deteriorate with increasing time, when mothers are asked about breastfeeding duration, the reports become increasingly inaccurate with increasing time since cessation [43,44]. As we assessed duration of breastfeeding both at a follow-up visit at 2 years and at 5 years, the time difference between the first of these visits and the time when many stopped breastfeeding was short, which probably limited recall difficulties. Lenore et al.[45] showed that infant feeding data collected within 18 months after the event can be used in epidemiological studies. Thus the 2 year recall breastfeeding in addition to the 5 year recall data could have enhanced the validity of mothers’ recall.

It is probable that respondents could have had preference for rounded and approximated answers (digit preference) for breastfeeding duration. This might have decreased the precision of the breastfeeding duration. There is a tendency to digit preference for example 2, 2.5 and 3 years among those who breastfed the longest and had not stopped breastfeeding at the 2 years visit. Still, it is less likely to be systematic differences between the groups. A Brazilian study [43], showed that mothers of higher social-economic status were more likely than their lower socio-economic counterparts to overestimate breastfeeding duration. With respect to the present study, the control group had more participants with high socio-economic status than the intervention group indicating a source of differential misclassification. However, adjusting socio-economic status in the multiple variable regression analysis did not lead to substantial change of the estimates.

Some would argue that using zero-inflated negative binomial regression analysis could be preferred to negative binomial regression analysis. A parallel analysis using this model showed similar results and thus did not change the conclusion of the study.

## Conclusion

The PROMISE EBF trial did not impact on early childhood caries or breastfeeding duration at 5 years of age. This study contributes to the body of evidence that promotion of exclusive breastfeeding does not raise oral health concerns. However, the high burden of caries calls for efforts to improve the oral health condition in this setting.

## Supporting Information

S1 Checklist (PDF)

S1 File Web appendix 2011. (PDF)

S2 File Histogram and box plot for early childhood caries. (PDF)

S1 Protocol PROMISE-EBF study protocol. (PDF)

S1 Table Results for zero inflated negative binomial regression. (PDF)
