# Supplementary material for: Effect of Breastfeeding Promotion on Early Childhood Caries and Breastfeeding Duration among 5 Year Old Children in Eastern Uganda: A Cluster Randomized Trial
Source: PLoS One. 2015 May 4;10(5):e0125352. doi: 10.1371/journal.pone.0125352 (PMC4418833; doi:10.1371/journal.pone.0125352)
Supplement: S1 File — (PDF) [file pone.0125352.s002.pdf]

THE LANCET

Supplementary webappendix

This webappendix formed part of the original submission and has been peer reviewed. We post it as supplied by the authors.

Supplement to: Tylleskär T, Jackson D, Meda N, et al, for the PROMISE-EBF Study Group. Exclusive breastfeeding promotion by peer counsellors in sub-Saharan Africa (PROMISE-EBF): a cluster-randomised trial. *Lancet* 2011; published online July 12. DOI:10.1016/S0140-6736(11)60738-1.

Web appendix for:

Exclusive breastfeeding promotion by peer counsellors in Sub-Saharan Africa: results from the PROMISE-EBF multicentre cluster-randomised behavioural intervention trial

Detailed PROMISE EBF Methodology and supplementary analysis

Thorkild Tylleskär, Debra Jackson, Nicolas Meda, Ingunn Marie S. Engebretsen, Mickey Chopra, Abdoulaye Hama Diallo, Tanya Doherty, Eva-Charlotte Ekström, Lars T Fadnes, Ameena Goga, Chipepo Kankasa, Jørn I. Klungsøyr, Carl Lombard, Victoria Nankabirwa, Jolly K. Nankunda, Philippe Van de Perre, David Sanders, Rebecca Shanmugam, Halvor Sommerfelt, Henry Wamani, James K. Tumwine, for the PROMISE-EBF study group.

Table of contents

A. Trial location ……………………………………………………………………………….. 2
B. Selection of clusters ……………………………………………………………………… 7
C. Randomisation …………………………………………………………………………… 8
D. Primary outcome measures and sample size …………………………………………………... 10
E. Development of data collection tools …………………………………………………………… 11
F. Criteria for inclusion, exclusion and termination ……………………………………………... 12
G. The peer counselling intervention ……………………………………………………………… 12
H. Follow-up ………………………………………………………………………………… 14
I. Electronic data collection with EpiHandy …………………………………………………….. 15
J. Supplementary analysis …………………………………………………………………… 21
K. References ……………………………………………………………………………….. 22
L. List of publications from the PROMISE EBF study to date …………………………………. 23

This document describes in greater detail the trial methodology that was used.

A. Trial location

The trial was carried out as a multicentre trial in 4 African countries, Burkina Faso in French-speaking West Africa, Uganda in East Africa, Zambia in Central Africa and South Africa. The Zambian site experienced floods in the middle of the trial and the results from this site cannot be analysed.

In each country a district was selected as the intervention site:

The **Burkina Faso** site was situated in rural Banfora, Cascades region, figure 1 (South-west of the country) where 76% of pregnant women attend antenatal care and 51% of deliveries were assisted by trained health staff. The under-5 mortality rate (U5MR) in Burkina Faso (2008) was 169/1000 live births and the infant mortality rate (IMR) was 92/1000 live births. The proportion of exclusive breastfeeding among children <6 months of age in Burkina Faso[1] 2005-2009 was estimated to 16%.

Banfora Health District covers an area of 15,000 km^2 and a population of 385,000. The district is divided into three administrative sub-districts: Banfora, Soubakénédougou and Sidéradougou. Farming and animal husbandry are the main sources of income in all three sub districts. The health care system consists of 60 primary health facilities (CSPS) and one regional hospital based in the town of Banfora. The main local languages are Dioula and Gouin/Karaboro.

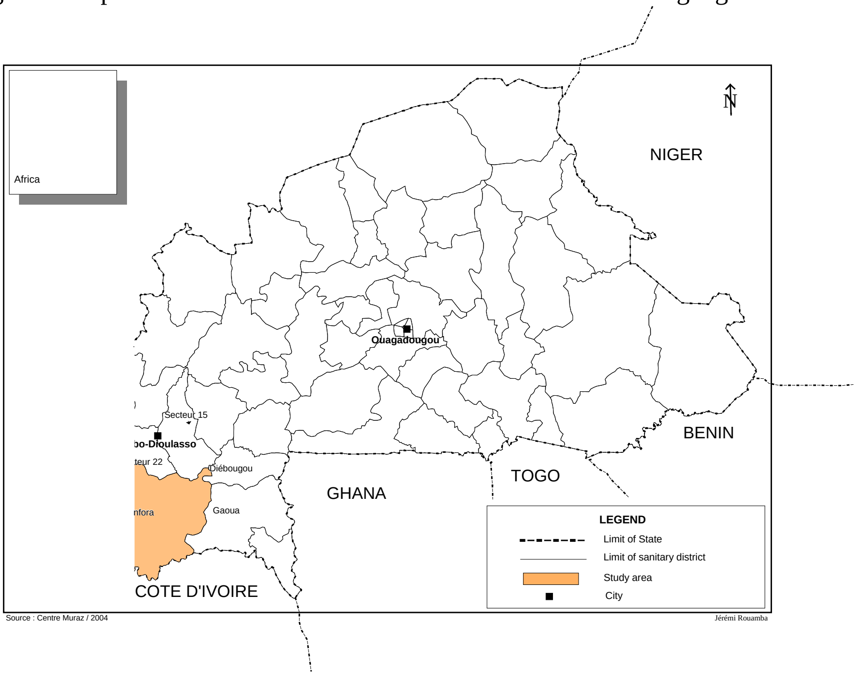

Figure 1a. In Burkina Faso, the trial site was located in the South-west, around and South of Banfora. Centre Muraz is located in Bobo-Dioulasso.

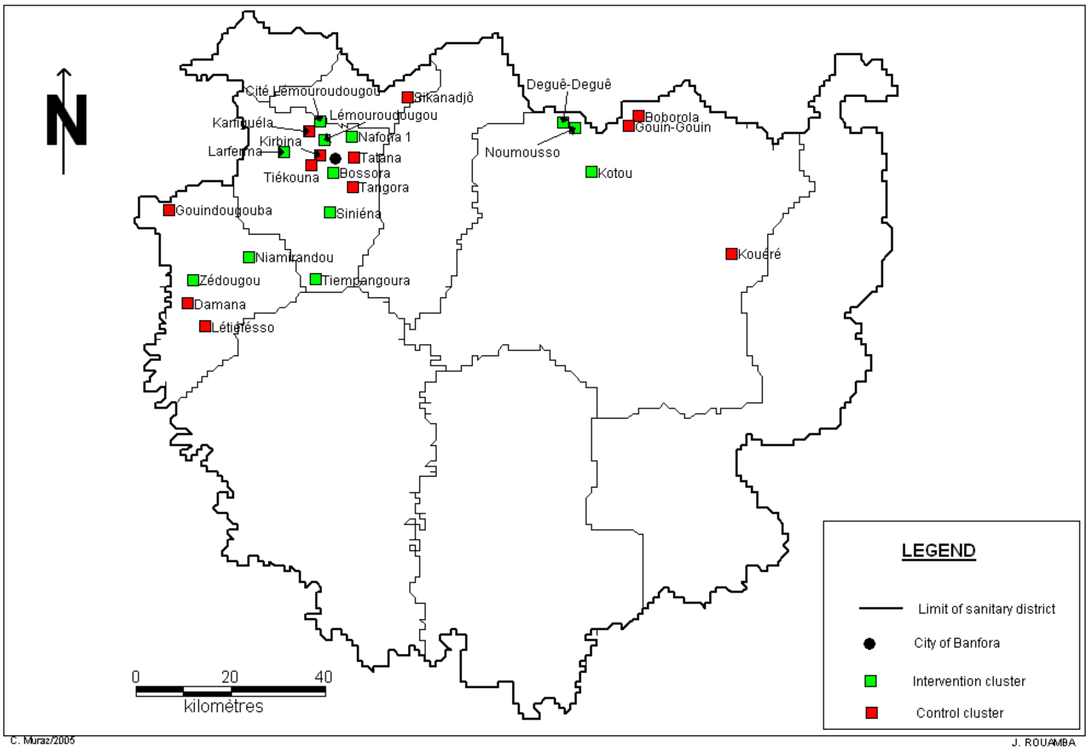

Figure 1b. The study area with intervention clusters (green) and control clusters (red) The city of Banfora is marked by a black dot.

The **Uganda**n site was Mbale District in Eastern Uganda, including urban Mbale Municipality and surrounding rural areas. Mbale Municipality is a trading centre, influenced by large slum migrant settlements. In 2006, 95% of the women attended antenatal care and 41% delivered in a healthcare facility in Eastern Uganda. The U5MR in Uganda (2008) was 135/1000 live births and the IMR in 2008 was 85/1000. The proportion of exclusive breastfeeding among children <6 months of age in Uganda[1] 2005-2009 was estimated to 60%, whereas nearly all mothers practiced breastfeeding. The regional HIV-prevalence among fertile women was 6.2% (2004-5).

Mbale District in Eastern Uganda, figure 2, is predominantly rural and has a population of 403,000 inhabitants and a population density of 535 per square kilometre. The study was done in two of the seven counties: the urban Mbale Municipality, situated approximately 230 km from Kampala, and the rural Bungokho. Mbale Municipality is the district centre and has approximately ten percent of the district population. Bungokho surrounds Mbale Municipality, and the population mainly consists of subsistence farmers. Mbale Hospital is both the District and the Regional Referral Hospital.

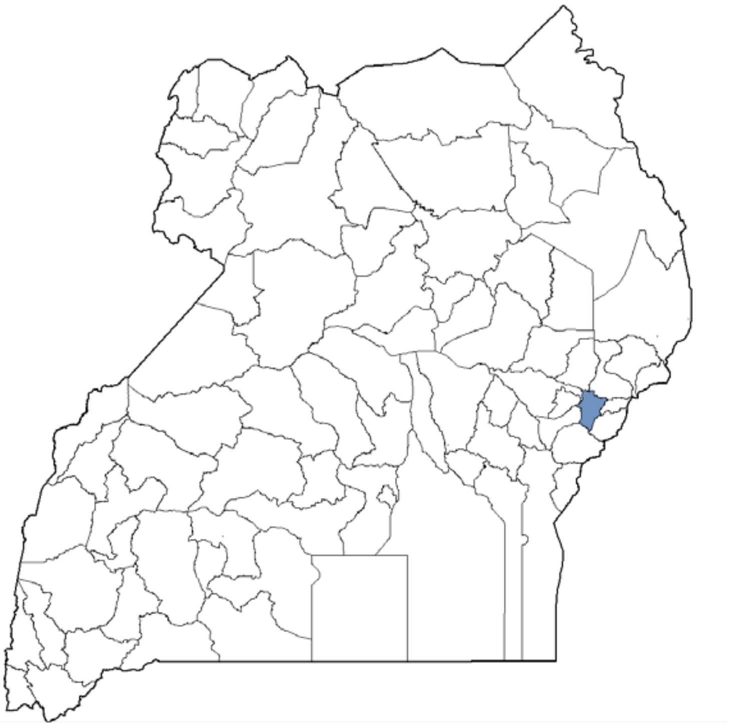

Figure 2a. The Ugandan site located in the Eastern part of the country, in and around Mbale.

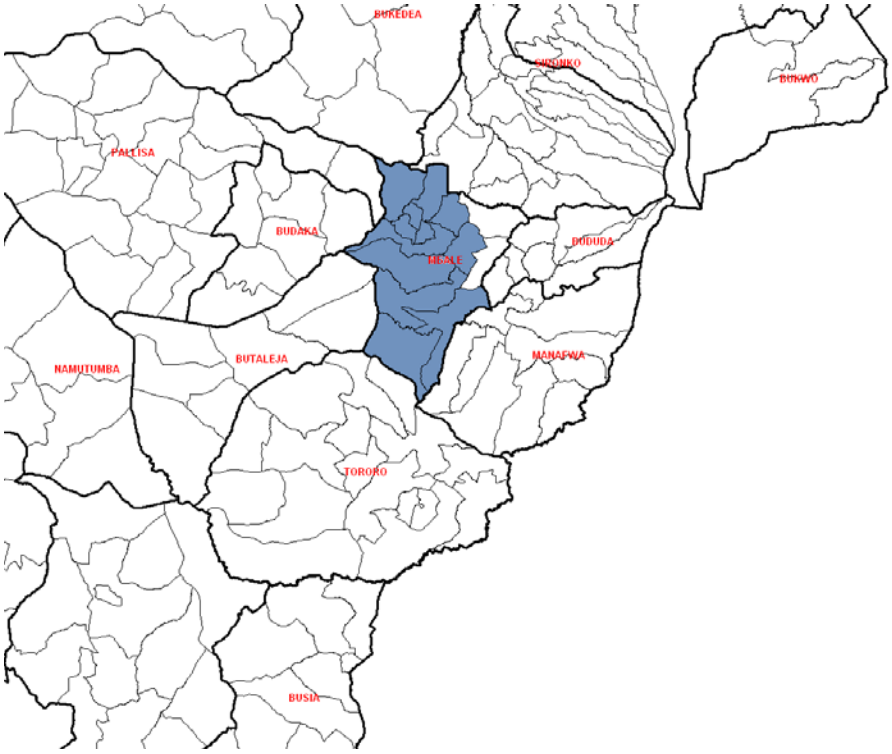

Figure 2b. Mbale District.

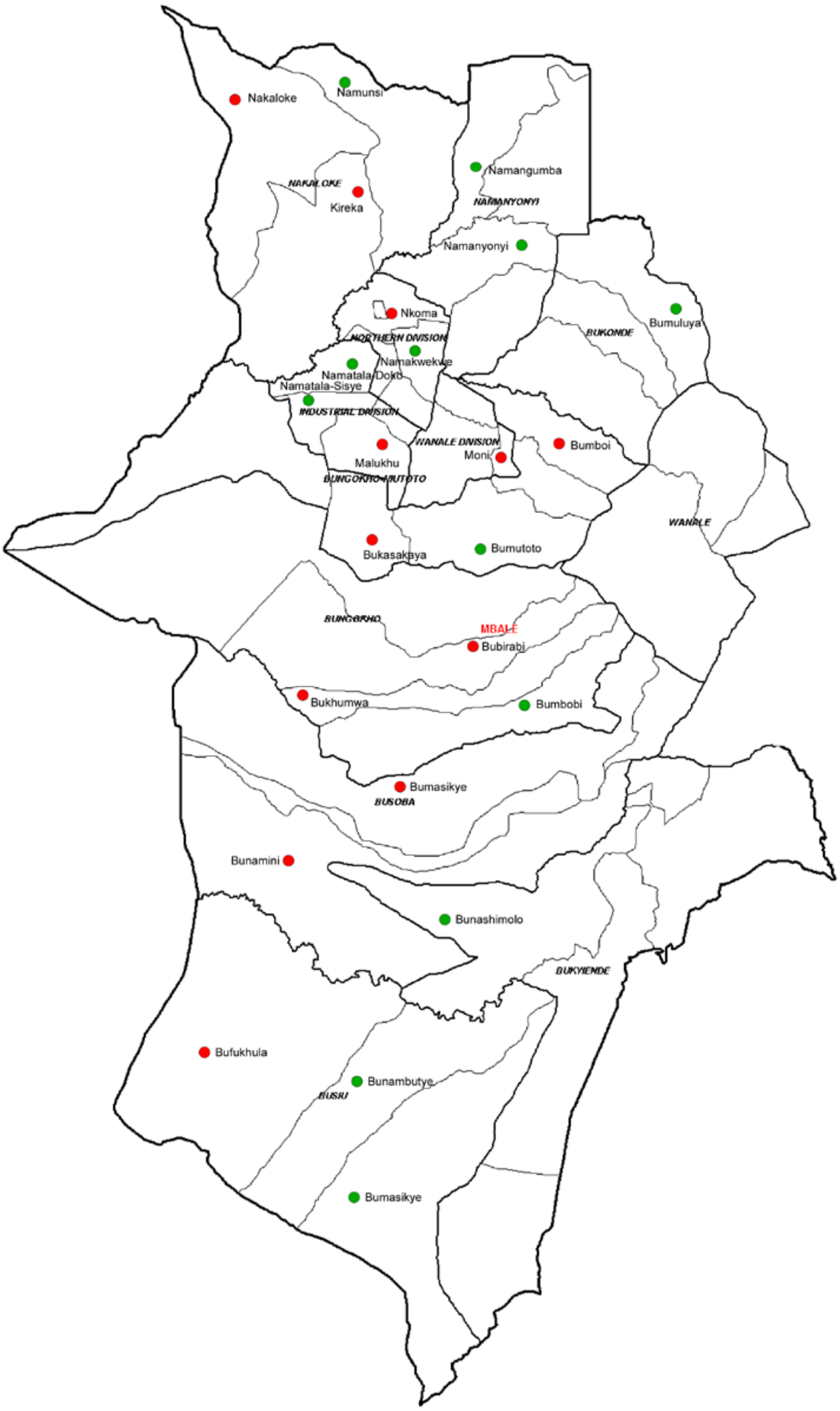

Figure 2c. The Ugandan site in Mbale District including adjacent rural Southern parishes with intervention clusters (green) and control clusters (red). The randomisation was stratified in urban Mbale Municipality (6 clusters in the mid-upper part of the map) and rural surrounding Bunghoko (18 clusters).

In **South Africa** 3 geographically separate sites were chosen, figure 3. Paarl, a town at the centre of a farming district close to Cape Town (Western Cape Province) and 2 sites in Kwa Zulu Natal Province: Umlazi (large periurban township near Durban) and Rietvlei (rural). Rietvlei is one of the poorest rural districts in South Africa. The U5MR in South Africa as a whole (2008) was 67/1000 live births and the IMR was 48/1000 live births. The local IMR and antenatal HIV-prevalence were: Paarl 40/1000 live births and 10%, respectively; Umlazi 60/1000 and 42%, respectively; and Rietvlei 99/1000 and 34%, respectively. Antenatal attendance and hospital delivery rates were 94% and 84% in South-Africa, respectively. The proportion of exclusive breastfeeding among children <6 months of age in South Africa[1] 2005-2009 was estimated to 8%.

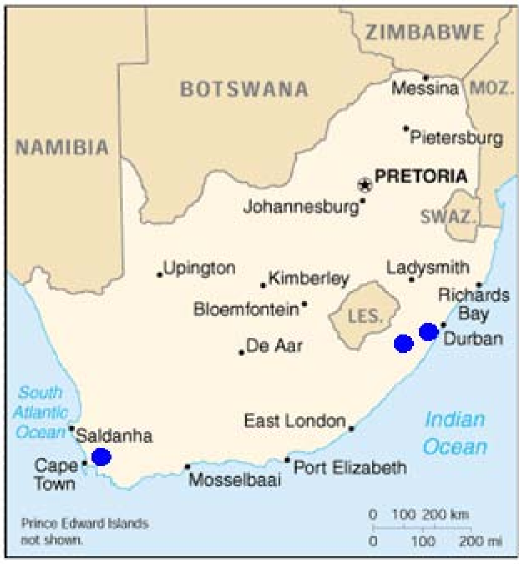

Figure 3a. In South Africa, the trial sites were located in Paarl (outside Cape Town), Umlazi (outskirts of Durban) and Rietvlei (between Durban and Lesotho).

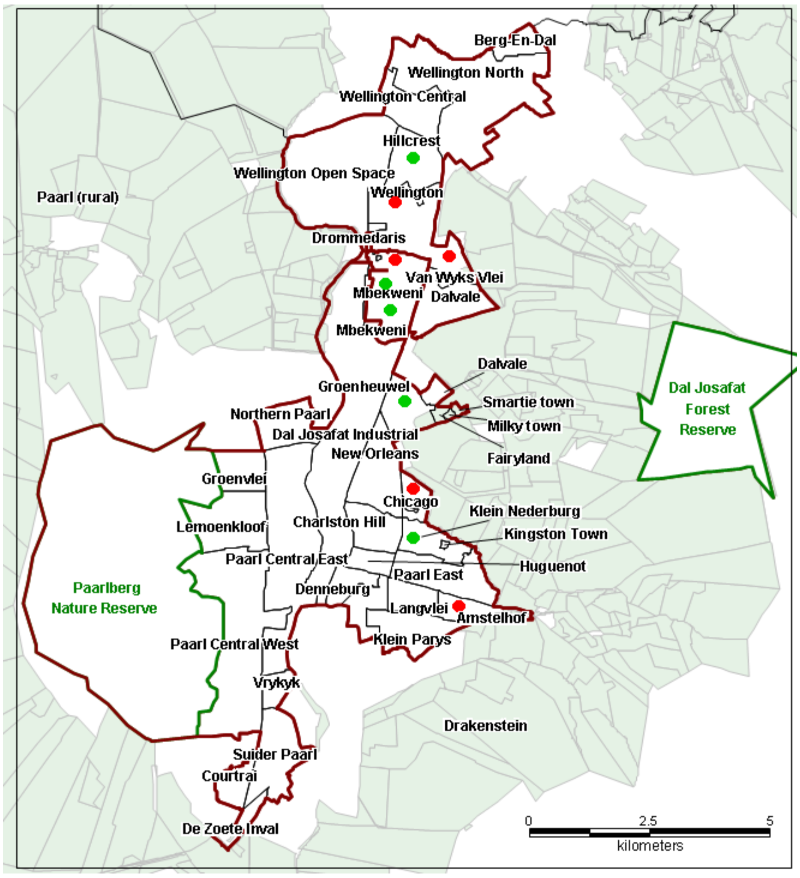

Figure 3b. The Paarl site outside Cape Town in the Western Cape Province with intervention clusters (green) and control clusters (red).

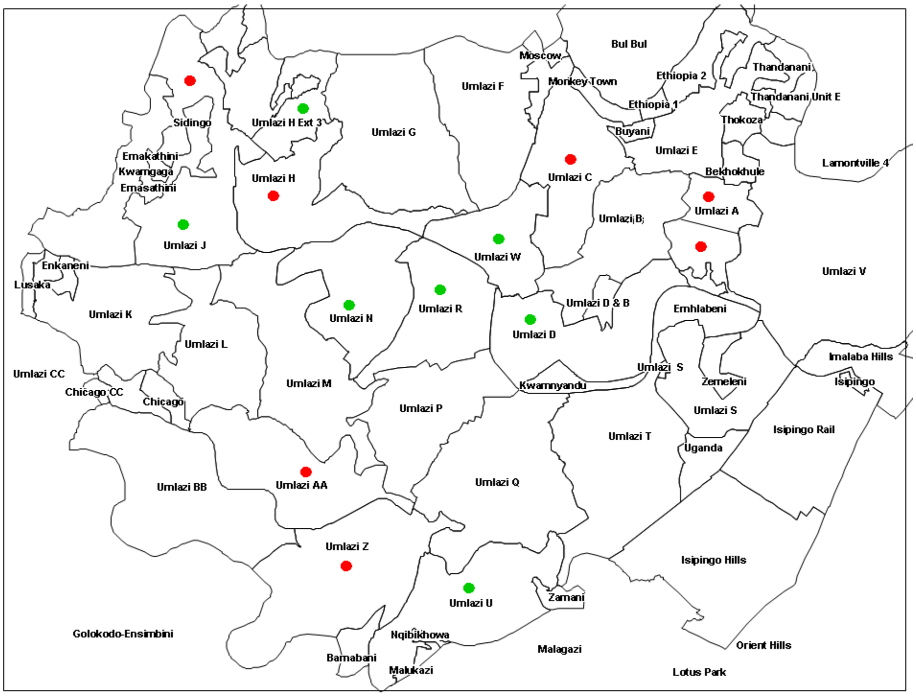

Figure 3c. The Umlazi site in peri-urban Durban, Kwa Zulu Natal Province with intervention clusters (green) and control clusters (red).

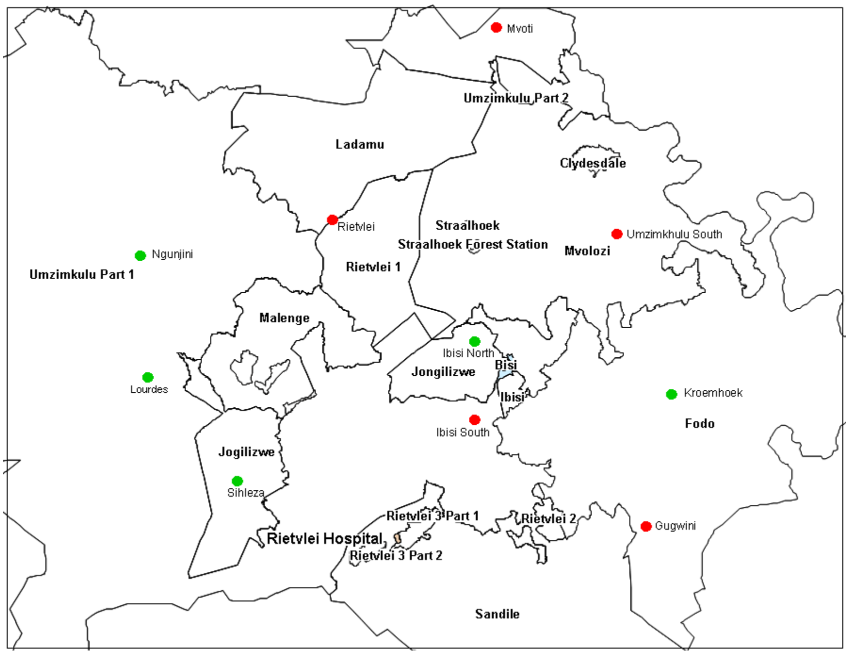

Figure 3d. The rural Rietvlei site south of Durban in Kwa Zulu Natal Province with intervention clusters (green) and control clusters (red).

B. Selection of clusters

Extensive information was given to community leaders, and the selection process of clusters was done in close collaboration with community leaders. Description of infrastructure (road, water, sanitation), facilities (health centres, schools, trading market and religious centres), geographical characteristics, and socio-economic and cultural characteristics (ethnicity, language) were provided before randomisation. Care was taken to allow for 'corridors' between selected clusters to be randomised. Statistical information from relevant administrative lists/national statistical bureaus or prior studies were used to estimate numbers and geographical sizes of clusters, yielding 24 clusters in Burkina Faso, 24 in Uganda and 34 clusters in South Africa.

C. Randomisation

The randomisation unit was defined as clusters and comprised 1-2 villages or communities with an average of 1000 inhabitants (~ 35 infants born per year, i.e. a birth rate of 3.5%).

The randomisation of clusters was country-specific. The site in Burkina Faso was treated as one single stratum. In Uganda, stratification was performed by urban/rural clusters, and by the 3 geographical distinct areas in South-Africa. In each stratum half of the communities were randomised to intervention and half to control. The allocation sequences for Burkina Faso and Uganda were generated by Halvor Sommerfelt and Rajiv Bahl and for South Africa it was generated by Carl Lombard.

Sequence generation
There was no stratification In Burkina Faso. Simple randomisation was used without any blocks. A list of pseudo-random numbers were generated in Excel 97 using the command "=rand()". The generated values were fixed by copying them as "values" next to the alphabetic list of the 24 preselected clusters. These were subsequently arranged in ascending order according to the generated pseudo-random numbers: and the first 12 were selected as intervention clusters, and the last 12 as control clusters.

The same procedure was used in Uganda, simple randomisation without any blocks. Within each of the 2 strata (rural and urban), a list of pseudo-random numbers were generated in Excel 97 using the command "=rand()". The generated values were fixed by copying them as "values" next to the alphabetic list of the 24 preselected clusters. The 18 rural clusters were arranged in ascending order according to the generated pseudo-random numbers. The first 9 were selected as intervention clusters and the last 9 as control clusters. Similarly, the 6 urban clusters were arranged in ascending order according to the generated pseudo-random numbers: the first 3 were selected as intervention clusters and the last 3 as control clusters.

In South Africa, the randomisation was stratified by site (Paarl, Rietvlei and Umlazi). Because 3 sites were involved, it the number of clusters was increased to a total of 34, (7 per arm in Umlazi, and 5 per arm each in Paarl and Rietvlei, giving a total of 17 intervention and 17 control clusters).

Allocation concealment and masking
Concealment of the allocation was not done for the participants, who inevitably got to know if they were in the intervention arm or not. Masking of the allocation was done for the data collectors by keeping the 2 teams for the intervention and the data collection separate, and avoiding informing data collectors about the allocation for the different clusters. However, there was a possibility that the data collectors learnt from the mothers or cluster visits whether they had been visited by someone talking about breastfeeding (= a peer-counsellor). The success of this masking was not formally evaluated.

Within-cluster recruitment and sampling
Pregnant women in the randomised communities were informed about the trial and asked if they wanted to participate. If they agreed, an enrolment interview was carried out from which the women was or was not accepted for data collection based on eligibility criteria. The women in the intervention clusters were offered the intervention irrespective of their participation in the data collection.

Informed consent was obtained from each woman prior to individual inclusion in the trial. This was a 2-stage procedure: the first step was verbal to obtain approval by the woman that she could be visited by a data collector for more information about the trial. The second was a written consent form, which applied only to women sampled for data collection. The data collector had to go through a comprehensive information sheet explaining all the trial procedures from inclusion criteria to the last follow-up. The women were allowed to ask questions and talk to their family (usually the husband). The data collector scheduled another visit a week later and then women who were willing to consent could either sign or put their finger-prints. If a woman declined, a form was filled and she was thanked. Irrespective of their participation in data collection, all women in intervention clusters benefited from counselling sessions on EBF if they wished.

Burkina Faso: recruitment and sampling
In each cluster (both control and intervention arms) community-workers, named ‘peer counsellors’ in the intervention arm (PC) and ‘recruiters’ in the control arm were recruited for the trial. They were trained and instructed to visit regularly each household in their village to enquire about pregnant women. Furthermore, they targeted specific places, like the market, water collection points and the mill, to identify pregnant women through friendly chats. Once a pregnant woman was identified, she was informed about the PROMISE EBF trial and was asked if she would like to know more about it. Her name was recorded along with the information of whether she was interested. The selection of women for peer counsellor and/or data collection participation was done in the following way in both arms of the trial.

A sampling frame was set up to secure equal chance of being selected for the trial. We included 4 women per cluster per month through random sampling, if there were more than 4 pregnant women in the cluster that month. To do so, a monthly meeting was scheduled in each cluster. A meeting was organized on an agreed date with all potential participants, and the names of all women fulfilling the inclusion criteria were noted on small identical pieces of paper. An independent person was invited to come and pick 4 names from the ballot. Because the consent approval rate was so high, we did not really feel it would be needed to pick more than 4 names, giving them all an equal chance of being selected. Instead of small pieces of paper in some clusters, we used a pot of sand where long and short sticks were embedded to the same height so that no one could identify the size before removing a stick. All eligible pregnant women (or husband or stand-in) were asked to come and pick one stick. Only those picking up the 4 long sticks were included in the trial. Each woman could only be included in the sampling once.

Uganda: recruitment and sampling
Participant selection was essentially similar in both arms of the trial. There was consecutive sampling in the Uganda site. All women identified in the clusters were included for data collection in the trial if they consented and met trial inclusion criteria. There was one designated appointed ‘recruiter’ of participants in each cluster. The recruiter usually had an important task in the community, like the head of the women’s committee. She was trained in trial procedures and confidentiality. Each time a recruiter identified a pregnant woman, she would talk to her about the trial. The recruiter would inform the trial office about the identified woman and the preferred day for the data collector to visit the woman, and also an appointment with the peer counsellor, if she was in an intervention cluster. The recruiter would introduce the data collector to the woman, who would seek the woman’s consent to talk before explaining to her the nature of the trial for which her consent was needed. The mother either signed or gave a thumbprint (if illiterate) in consenting. All the signed forms were filed at the trial office. However, when a woman was unwilling to participate in the trial or did not meet the inclusion criteria, but was nevertheless interested in having counselling, the peer counsellor continued to visit her. In case of withdrawal, the woman’s decision was respected and the reason noted when possible.

South Africa: recruitment and sampling
In two of the sites (Umlazi and Paarl) pregnant women were identified using a census approach. This entailed the peer counsellor visiting systematically every third house starting from his/her own house. At the house the counsellor would ask about pregnant women and women of childbearing age in the residence and in the neighbouring houses. This process allowed the identification of pregnant women and women of child bearing age. The pregnant women were recruited immediately if they were in their last trimester, or scheduled for a repeat visit in case of early pregnancy. Households with women of child bearing age were visited every quarter to identify any new pregnancies. In the third site (Rietvlei), pregnant women were identified through a snowballing approach by word of mouth and informal meetings in the streets. The peer counsellors also visited antenatal clinics to recruit women. Women who agreed to peer counselling were automatically included in the list for trial participation. For women who declined peer counselling, details were taken of their home address, if agreed, and this information was passed on to the data collection team for trial participation. Hence inclusion was not dependent on participation in the intervention. In order to have a random sample of all women in the cluster, a master list per cluster was established with information about who was receiving peer counselling, who was participating in data collection, who had delivered, dropped out, declined participation, etc. This list was only available to the supervisors wishing to track women. In brief, the sampling procedure was that the peer counsellor supervisor drew up each month a list of new women identified in the previous month. This list was given to the data collector supervisor for sample selection. Sampling was done one cluster at a time to recruit 3 women per cluster per month with 1-2 back-ups in case of non-participation. Data collectors visited the women selected. They introduced themselves and the data collection component of the trial, and obtained initial informed consent. After eligibility for the trial had been assessed, the mother meeting the criteria and willing to participate went through a full informed consent procedure.

The data collectors asked the mothers if they had undergone counselling and HIV testing at the antenatal clinic, and whether they were willing to disclose their test results. Mothers who were known to be HIV-negative or unwilling to disclose their result were treated as HIV-negative or of unknown status for the trial, and were followed to 24 weeks. Mothers who disclosed that they were HIV positive were recruited into another parallel study. If the mother has not been HIV tested, she was encouraged to visit the antenatal clinic, and the matter of her HIV status was brought up again at a subsequent visit.

Potential selection bias due to cluster design or other aspects of the trial.
In order to minimise selection bias in this cluster-randomised trial,[2] all pregnant women in the randomised clusters were identified and approached. The likelihood of the study team missing a pregnant woman was very small. And in addition, only 52 of the women (2%) declined participation. This makes it unlikely that selective recruitment contributed importantly to the observed effect sizes.

D. Primary outcome measures and sample size calculation

The primary outcome measures of the trial were:

- **EBF prevalence at 3 months.** In the intervention group receiving peer counselling on EBF for 6 months, a rise in the proportion of infants exclusively breastfed from birth through 3 months by 20 percentage points from a baseline of 20% was expected. The 3 month EBF prevalences were to be country specific.
- **Decrease in diarrhoea.** In the intervention group, we expected a reduction of one-third from an estimated baseline of 12% diarrhoea incidence in the last 2 weeks to 8% in the intervention group. The impact of increased EBF on infant morbidity required a larger sample, the size of which was calculated for the whole trial.

Table 1. Assumptions used for the sample size calculations in addition to 1:1 randomisation and equal cluster size.

		Increase in EBF from 20% to 40%	Decrease in diarrhoea from 12% to 8%
Proportion in the intervention group	p_1	0.4	0.08
Proportion in the control group	p_2	0.2	0.12
Percentage point for alpha error = 0.05	z_1	1.96	1.96
Percentage point for beta error = 0.20	z_2	1.28	1.28
Number of individuals in each community	n	35	35
Coefficient of variation of proportions among clusters in each arm	k	0.4	0.3
Average of P1 and P2	p	0.3	0.1
Number of communities needed per arm	c	12	47

We based our sample size calculation on the expected reduction in cases of diarrhoea (at the age of 3 months) over the last 2 weeks of one-third from 12% to 8%. With a 95% confidence (alpha error 0.05) and power of 80%, and an average number of infants of 35 per randomised community, and a coefficient of variation between the communities of 0.3, we needed to randomise 48 communities in each arm – a total of 96 communities (Table 1). The sample size was based on the following formula:[3]

$$c = 1 + \frac{(z_1 + z_2)^2 \left[2p(1-p)/n + k^2\left(p_1^2 + p_2^2\right)\right]}{\left(p_2 - p_1\right)^2}$$

Table 2. Number of clusters needed per arm for different scenarios with a 95% confidence (alpha error 0.05) and power of 80% (beta error 0.2), at given number of participants per cluster and given intracluster coefficients of variation of the proportion of EBF or diarrhoea in each arm. The grey areas indicate which scenarios were covered by the sample size selected initially.

	Coefficients of variation of proportions among clusters in each arm[a]						
	0.20	0.25	0.30	0.35	0.40	0.45	0.50
EBF increase from 20% to 40%							
- 30 participants per cluster	7	8	10	11	13	16	18
- 32 participants per cluster	7	8	9	11	13	15	18
- 35 participants per cluster	7	8	9	11	12	15	17
- 38 participants per cluster	6	8	9	11	12	15	17
EBF increase from 35% to 70%							
- 30 participants per cluster	5	6	7	9	11	13	16
- 32 participants per cluster	5	6	7	9	11	13	16
- 35 participants per cluster	5	6	7	9	11	13	16
- 38 participants per cluster	5	6	7	9	11	13	16
Diarrhoea decrease from 12% to 8%							
- 30 participants per cluster	46	49	53	57	63	68	75
- 32 participants per cluster	44	47	51	55	60	66	72
- 35 participants per cluster	41	44	47	52	57	63	69
- 38 participants per cluster	38	41	45	49	54	60	67

[a]The intracluster coefficients of variation obtained in the study are presented in table 5 in this appendix.

This sample size, 12 clusters per arm in each country, should have accurately given us the country-specific estimate for the increase in EBF, and the 48 clusters per arm across all countries would allow us to document the above decrease in diarrhoea morbidity. No sample size calculation programme was used in the process.

The sample size depends on the number of individuals participating in each cluster and on the coefficient of variation of the proportions in each arm. Table 2 presents the sample size in a sensitivity analysis of these two factors.

Drop-outs/loss to follow-up was catered for in the sample size calculation by using coefficients of variation towards the higher end of the estimated spectrum, both for EBF and diarrhoea. In addition, to cater for the fact that 3 different sites were used in South Africa, 10 clusters were added in South Africa, totalling 34 clusters.

E. Development of data collection tools

The development of the questionnaires was guided by a review of the following documents.
Scientific background:

- Piwoz E. Breastfeeding and replacement feeding practices in the context of mother-to-child transmission of HIV. An assessment tool for research. WHO/RHR/01.12, WHO/ CAH/01.21. Geneva, Switzerland, World Health Organization, Department of Reproductive Health and Research (RHR), Department of Child and Adolescent Health and Development (CAH); 2001
- Bland RM, Rollins NC, Solarsh G, Van den Broeck J, Coovadia HM. Maternal recall of exclusive breast feeding duration. *Arch Dis Child* 2003;**88**:778-783.

A separate infant feeding and HIV cohort study in South Africa, known as the Good Start study, a cross-sectional study in Mbale, Uganda conducted in 2003 and a methodological infant feeding study from 2005 helped in the development of the PROMISE EBF questionnaires. These studies lead to the following publications:
1. From Good Start:

- Doherty T, Chopra M, Jackson D, Goga A, Colvin M, Persson L-A. Effectiveness of the WHO/UNICEF guidelines on infant feeding for HIV positive women: results from a prospective cohort study in South Africa. AIDS 2007;21:1791-7
- Goga AE, Van Wyk B, Doherty T, Colvin M, Jackson DJ, Chopra M, Good Start Study Group. Operational effectiveness of guidelines on complete breast-feeding cessation to reduce mother-to-child transmission of HIV: results from a prospective observational cohort study at routine prevention of mother-to-child transmission sites, South Africa. *J Acquir Immune Defic Syndr* 2009;**50**:521-8.

2. From the formative Mbale infant feeding study (Uganda):

- Engebretsen IM, Tylleskar T, Wamani H, Karamagi C, Tumwine JK. Determinants of infant growth in Eastern Uganda: a community-based cross-sectional study. *BMC Public Health* 2008;**8**:418.
- Engebretsen IM, Wamani H, Karamagi CA, Semiyaga N, Tumwine JK, Tylleskar T. Low adherence to exclusive breastfeeding in Eastern Uganda: a community-based cross-sectional study comparing dietary recall since birth with 24-hour recall. *BMC Pediatr* 2007;**7**:10.
- Engebretsen IM, Shanmugam R, Sommerfelt AE, Tumwine JK, Tylleskar: Infant feeding modalities addressed in two different ways in Eastern Uganda. *Int Breastfeed J* 2010;**5**:2.

Dr Haider from India, the principle investigator on a large randomised trial of peer counselling for EBF in India was consulted. She also gave some input on questionnaire development, especially feeding questions. Uganda DHS was contacted to assess how infant feeding questions were asked in that national survey. Experts at WHO, especially Dr Rajiv Bahl, gave input on the development of the questionnaires.

The aim of the recruitment questionnaire was to collect important background information about the population under study and important potential confounders. The aim of the post-partum questionnaires was to gather information to assess the trial outcomes. The design of the trial made it possible to have a set of core-questions common to all the post-partum questionnaires. We aimed at making all questions as neutral/objective as possible to avoid sensitizing the mothers towards the intervention.

Socio-economic status was assessed by creating an index with the use of Multiple Correspondence Analysis, MCA, from a set of wealth items asked in each individual country. Each country specific wealth index was divided into quintiles. (MCA belongs to the group correspondence analysis, CA). CA is a method of factoring categorical variables and displaying them in a property space that maps their association in 2 (CA) or more dimensions (MCA). The reason for this choice was to take into account that the vast majority of the variables being analysed were of a categorical nature, and more commonly of a dichotomous nature (either a “yes” or “no” response). Over the last decade, wealth assessment has been captured by PCA (principal components analysis) or FA (factor analysis) in settings where income and consumption-based data has been hard to retrieve. This was described by Filmer and Pritchet[4] and used by the

Demographic and Health Surveys. The major features of these techniques are that, where conventional PCA and FA analysis determines which variables cluster together, correspondence analysis determines which category values are closer together. This is visualized on the correspondence map, which plots points (categories) along the computed factor axes. This is essentially the aim of most SES analysis – the aim is not to cluster variables together (the researcher has already set up the questions so that he/she knows they are markers for SES and so are in a sense already clustered), but to cluster together the categories within these variables. To this end it made more sense to employ a CA or, in our case, MCA rather than the PCA/FA more commonly used.

For the infant feeding recalls we used 1) a 24-hour recall, asking the mother if she breastfeed from yesterday morning till this morning and assessing if the baby had got any other items from a 22-item list, including an unspecified option; and 2) a 1-week recall, asking the mother if she breastfeed last week, ending yesterday morning, and assessing if the baby had got any other items from a 22-item list, including an unspecified option. For diarrhoea, we had a 24-hour recall and a 2-week recall.

In the development of data collection tools care was taken to prevent bias, particularly as the outcome was self-reported. A number of potential recall biases have been described:[5] recall bias due to too long recall period, selective recall, social desirability, interview situation and interviewing tools, question phrasing, poor answer alternatives and digit preference.

F. Criteria for inclusion, exclusion and termination

The inclusion and exclusion criteria for pre-inclusion (the recruitment interview) were as follows:

1. Live in trial area and not planning to move within trial follow-up period
2. Signed informed consent (minimum age for legal consent varies by country)
3. At least 7 months pregnant (or visibly pregnant if the estimated date of delivery was not known)
4. Intending to breastfeed. In Burkina Faso and Uganda this implied operationally that women who had opted for exclusive formula feeding as part of a positive HIV-test counselling would not be included in the study. At the time of the study, provider initiated opt-out counselling and testing was not initiated, and therefore the known HIV-positive prevalence was very low. Without a clear medical indication, e.g. HIV-positive status, women would have been encouraged to breastfeed as part of the antenatal care counselling. South-Africa had a different structural set-up with regard to HIV, and women were counselled on ‘exclusive’ feeding. Those HIV-positive women opting for exclusive breastfeeding and not exclusive formula feeding were included in the PROMISE EBF trial.

After the birth of the child the additional criteria for inclusion were:

5. Singleton live birth
6. No serious infant congenital malformations or other infant illness that would preclude EBF, e.g. neonatal intensive care admission or extended neonatal hospital stay

The following termination definitions were used:

- *Withdrawal* – enrolled in trial, but later declines to participate or withdraws from trial. Moved – woman or infant moves out of trial area and can no longer be followed up. Note: If moved within trial area in same or different clusters – stays with original arm allocation and can be continued in data collection.
- *Loss-to-follow-up* – Cannot locate mother and baby and/or don’t know where they went/what happened to them. Peer counsellors were to continue to monitor if mother/infant returns and retain in trial if found. For data collection, follow-up visits are made 3 times per data collection visit before classifying as missed visit. In this category, if original address was confirmed and at least one data collection visit made, follow-up visits were supposed to be continued through 24 weeks in case mother returned to the address and only after this was the mother/infant pair classified as lost. Timing of loss was counted as first lost visit.
- *Maternal death* – mother dies
- *Infant death* – infant dies

Note: If a mother OR infant died, that pair were excluded from further follow-up. They were counted in the trial profile as a termination under the one that happened first if both occurred.

G. Peer counselling intervention

A Facilitators and Participants Guide was developed based on the WHO/UNICEF Breastfeeding counselling: a training course: http://www.who.int/child_adolescent_health/documents/who_cdr_93_3/en and the HIV and infant feeding counselling courses: http://www.who.int/child_adolescent_health/documents/9241592494/en/index.html. These courses were simplified and shortened to meet the needs of lay counsellors. The curriculum used the proven methods proposed in the above courses and covered all the essential aspects of breast milk production and supporting breastfeeding women. A field guide was also developed summarised the course content and was given to lay counsellors to ensure consistent high quality counselling.

The core components of the curriculum included sessions on:

- Your own beliefs about feeding
- Communication and counselling skills
- Composition of breast milk and the importance of breastfeeding
- How milk is produced and released by the breast (frequent feeds increases milk production)
- Helping a mother with positioning herself before she starts breastfeeding
- Attachment of the baby to the breast
- Breast conditions
- Expressing and storing breast milk
- Normal stools and urination
- Practicing exclusive breastfeeding
- Common feeding difficulties (how to handle a crying baby)
- Role plays
- Clinical practice sessions

During each of the visits the peer counsellors undertook the following:

- Provide support, help and counselling to women to optimize their infant feeding practice (as stated in the box above),
- pre-empted and discussed common feeding difficulties
- addressed the mother's/family's feeding concerns/difficulties
- provided basic relevant information on the importance of immunisation and other child health issues
- If the need arises, peer counsellors identified those mothers or children needing urgent referral to the clinic.

The content varied according to the counselling visit. The antenatal visit focused on initiation, colostrum and EBF. Unscheduled peer counsellor visits (at the request of the mother) were allowed.

In South Africa only, the importance of 'exclusive' feeding (breast or formula) to reduce the chances of mother-to-child transmission of HIV was taught to the peer counsellors. As mentioned earlier, HIV-positive women were only included in the PROMISE EBF trial if they opted for breastfeeding. Counselling on 'exclusive feeding' if HIV-positive was the responsibility of the health units and not the peer counsellors and the PROMISE EBF trial included those opting for breastfeeding.

The peer counsellor counselling package was piloted in a joint training across all sites using South African peer counsellors. This experience was used to improve the programme. Updates and repeated training of peer counsellors were done when gaps were identified and new peer counsellors were recruited.

The peer counselling package was used to train peer counsellors in Uganda and Burkina Faso. Emphasis was put on exclusive breastfeeding counselling in these countries, as the peer counsellors alone were supporting mothers for exclusive breastfeeding. The peer counsellors were followed up fortnightly by supervisors, and each peer counsellor was observed monthly to obtain feedback and improve counselling skills. In addition, the supervisors organised monthly group meetings with the peer counsellors. The women were supposed to be visited by the peer counsellor once during the antenatal period, and at 1, 4, 7 and 10 weeks post-delivery. Mothers with breastfeeding problems were offered extra visits, as well as mothers who so requested them. A visit was declared 'missed' if the peer counsellor failed to find the mother at home on 3 different occasions during the week scheduled for the visit.

In Burkina Faso, peer counsellors were selected by the community themselves after 2 community-meetings held in each cluster. During the first meeting, the trial team met with community leaders, explained the trial objective and asked them to inform all the women in the village and schedule another community meeting. During the second meeting, the community itself set the criteria for a good peer supporter and designated 2-4 women for the task. In a third stage, the trial team selected the best candidates based on formal and personal criteria.

In Burkina Faso, all the peer counsellors had motherhood (including breastfeeding) experience based on the community selection; their mean age was 30 years. Three quarters of them could write (French or the native Dioula language) and no one had more than 2-6 years of primary schooling. Although attempted, this latter point made it difficult to get written reports from the peer counsellors in Burkina Faso. The one-week training course to the 28 peer counsellors in Banfora health district was provided by 4 supervisors with background in gynaecology-obstetrics, midwifery/nursing, sociology and clinical research. No peer counsellor among the 28 resigned. Although written reports from the peer counsellors were scarce, study team assessments of the intervention revealed good knowledge among them.

In Uganda, the trial team invited the village local council chairpersons from all selected intervention clusters to a meeting in Mbale town, where they were informed about the trial. Each of the chairpersons subsequently organized a meeting with women in their respective villages. At these meetings, women were informed about the trial and asked to identify women for training as peer counsellor for breastfeeding. The women proposed 2-3 candidates who were

interviewed by the trial team which identified the most suitable woman for training. The selected woman was announced to the meeting, which then accepted her as their representative to be trained as their peer counsellor. Twelve women were selected, one from each of the intervention clusters.

To be selected, a woman had to be between 18 and 45 years and to reside in the area with no plans of leaving the area within 2 years. She had to have a good reputation in the community. Furthermore, she had to be literate in the local language, willing to participate in the trial including a one-week residential training, and to undertake home visits in order to help women breastfeed their babies. Previous personal experience of breastfeeding was an additional advantage, although not a strict inclusion criterion.

The 12 women completed the training. Their age range was 25-40 (average 34 years). All had attained at least 7 years of formal education; one had obtained a diploma in secretarial studies after 11 years of formal education. Eleven were married, and most were full-time subsistence farmers and child carers. All had breastfed their babies, except one, who had not yet had a baby of her own.

The methods used in the training included lectures, small group discussions, plenary discussions, role plays and hands-on practice with mothers who had just delivered at Mbale Regional Referral Hospital. The peer counsellors were also taught how to complete the peer counsellor visit forms and record information at each visit. This information included dates of peer counselling visits, the duration of a counselling session, and a checklist of topics discussed with the mothers. During training, the proper timing of peer counsellor visits and the key messages to share with the mothers during different visits were emphasized. Peer counsellors were advised to counsel and support all pregnant mothers identified within their clusters.

In South Africa, the selection of peer counsellors was a step-wise process and only females were considered. The trial team had a more central role identifying the women in South Africa compared to Uganda and Burkina Faso. Personal, formal and practical criteria were set in the selection process: 12 year of schooling, owning a bank account, good reputation in the community, previous community involvement and personal positive characteristics (empathy, patience, etc.) Formal tests and an interview were conducted prior to an observation during role plays. A one-week course was held where South Africa-specific procedures and the curriculum was taught. They also had training on filling the forms for their counselling visits.

Each peer counsellor visited the same woman each time to ensure continuity and the development of a trusting relationship between them. The peer counsellor visit record form was used to document visits. In addition, mothers kept a home-held form to record when they were visited by the peer counsellor. If a mother was not at home, the peer counsellor tried to visit the mother again during the week in which that visit had been scheduled. If the peer counsellor had made 3 visits during the week that the visit was scheduled and had not been able to locate the mother, this was classed as a missed visit. One supervisor was employed for 10-12 peer counsellors, whose role was to provide support and encourage high quality consistent counselling.

H. Follow-up

Mother-infant pairs were followed up from the recruitment interview antenatally to the final visit scheduled at week 24 after birth. The schedule for data collection visits was weeks 3, 6, 12 and 24. In case of a missed appointment, the data collector was instructed to inform the participant family and give them a tentative date for another visit. The data collector had to attempt to see a woman at least 3 times within the normal trial range before he could record a missed visit. In the case of maternal or child death, a verbal autopsy form was filled whenever possible and the trial was terminated.

In Burkina Faso, data collectors were recruited through newspaper advertisements and a 2-step procedure, which included a written test and an interview. Seven candidates were selected, 4 men and 3 women. They had completed secondary school and previously worked in a research trial from a period of 1-5 years. The data collectors got a 5-days training workshop and a 2-days field testing of both paper-based questionnaires and the electronic data collection tools. Furthermore, they were given refreshment trainings and supervision. Data cross-checking was conducted. An information system was set up with the peer counsellors/recruiters to collect the names of pregnant women in each cluster on a weekly basis, know the women who had given birth and when, and identify infant or maternal deaths when they happened. Each participant had a trial yellow card with her inclusion number and names for easy identification by the peer counsellor/recruiter. Regular supervision and re-training of data collectors was performed. This included observations, cross-checking of data and meetings.

In Uganda, 7 individuals, 2 men and 5 women (aged 20 to 30 years) were identified for the job as data collectors through a similar process as in Burkina Faso. They were fluent in English and Lumasaaba (the local language) and had at least a bachelor degree A-level, basic computer knowledge and previous work experience. Most of them had

previously been involved in data collection. A one-week training workshop was held at the start-up of the trial, including training on the standard operating procedures (SOPs) and ethics. Each data collector was given a manual which were reviewed during the training. A refresher training workshop was held 9 months after the start of data collection. The agenda and topics covered were similar to those in the original training programme. In addition, common challenges were reviewed and issues clarified.

In South Africa, each of the 3 trial sites already had data collectors from previous studies. One data collector also acted as an on-site data collector supervisor who liaised with the peer counsellor supervisor to obtain monthly lists of the enrolled women. A field manager visited all sites regularly to provide support and supervision to the teams, data collection and general site issues.

I. Electronic data collection with EpiHandy

Section written by Jørn Klungsøyr, Hama Diallo and Thorkild Tylleskär

This section describes the EpiHandy software and how it was implemented in Uganda and Burkina Faso as an electronic data collection and management solution for the Promise EBF study.

EpiHandy is a set of tools for electronic collection and handling of many types of data on mobile devices for the entry of data directly in an electronic format. EpiHandy installation tools, manuals and source codes are available for download from www.epihandy.org. EpiHandy was initially developed at the Centre for International Health, University of Bergen, Norway, in response to problems faced in the management of relief operations/research in remote rural areas of Ethiopia. Since the implementation of EpiHandy in the Promise EBF study, the lessons learned and improvements became the basis for a completely new and rewritten upgrade of the EpiHandy software called "openXdata", which can also be loaded on regular mobile phones (more details can be found at: www.openxdata.org).

Major components of EpiHandy

EpiHandy Server is a set of tools and solutions that handles storage of both forms and data. It is built around the .NET Framework and relies on Microsoft Desktop Engine 2000 (MSDE 2000) for industry standard database storage and data management. The hardware requirements are a Pentium 300Mhz computer with 256Mb memory and 10GB storage disk.

EpiHandy StudyManager is a desktop application that allows researchers to design and manage multi-lingual questionnaires that control the data collection activity in the field. There is no coding needed and wizards guide the process of setting up advanced validation rules, skip patterns/branching, translation of the questionnaires, and export of collected data in many common formats, including SPSS

The multi-language features enable design and setup of a form in a study once, which can then be translated it into any number of languages, without affecting the structure of a survey. A whole survey can be duplicated by a drag-and-drop procedure.

The designed forms can be previewed and tested in the StudyManager, or exported to a text or Word document for easy printing and proof-reading. EpiHandy StudyManager can also be used to design paper-based questionnaires through the above feature. There are basically no restrictions in the number of different questionnaires, pages, questions or options of a question that a study form can contain.

EpiHandy MobileClient is the tool used to collect responses to forms, which has 2 main purposes. One is to open the downloaded questionnaire and act as the interface towards the interviewer. And the second is to store the entered data in the questionnaire with a unique identifier and act as the interface towards the EpiHandy Server database, that holds all the collected data for the study. This is a small piece of software specially designed for handheld computers which runs on Windows Mobile 2003 or later version with .NET Framework.

Filling in a form on the EpiHandy MobileClient is done by tapping on the screen to answer the question. The user interface (the screen) of the EpiHandy MobileClient is responsive, with a structured easy-to-navigate layout. The user can move forward and back through question in a form. This has been made to reflect closely the freedom of pen and paper, whilst still ensuring consistency through data input types, validation and branching logic. Users can collect data for days at a time before coming to the office to upload data, the limiting factor being the memory capacity of the mobile, a maximum of 2GB (Gigabyte), which is plenty for studies where human beings will key in responses directly.

EpiHandy MobileClient - Components

EpiHandy main menu: After data collectors start the handheld computer, they will be presented with the main menu from which they can choose to open a form, synchronize forms to/from the server, do some simple troubleshooting and change default settings.

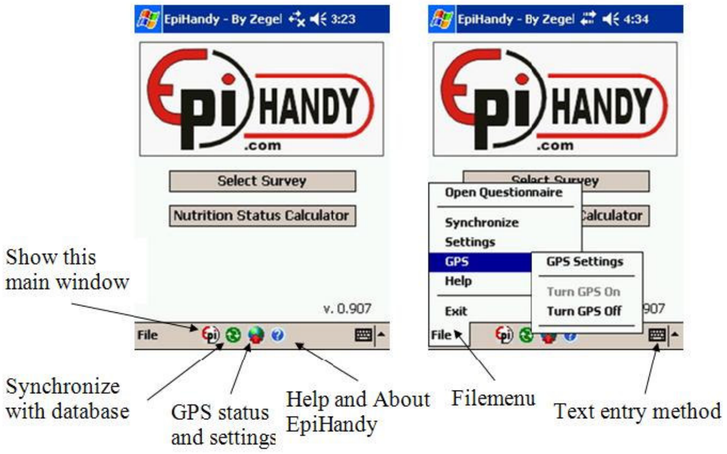

Figure 4. EpiHandy Main menu annotated.

EpiHandy form viewer: After a user has selected a form to open the viewer, it will be displayed using the structure and layout, figure 5. In this window the user can click on any question to start filling in information, scroll back and forth between pages, open the GPS viewer, and save and close the form viewer.

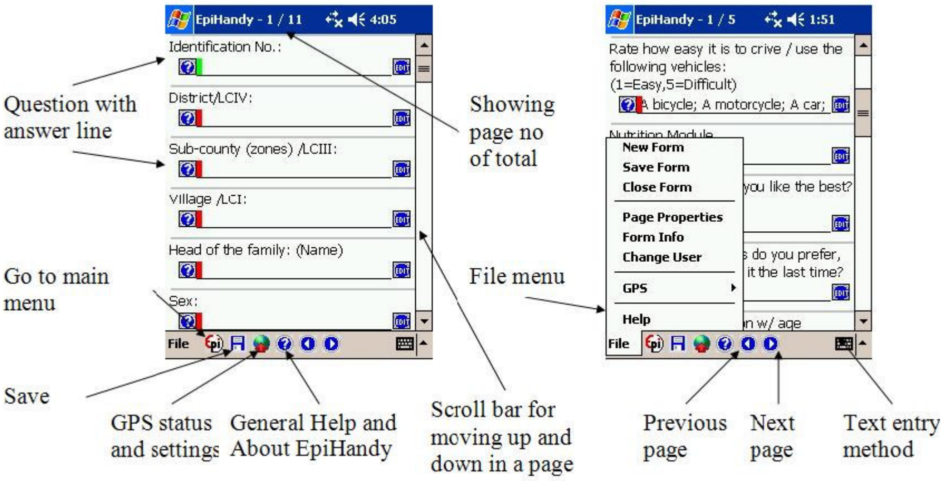

Figure 5. EpiHandy FormViewer and menus annotated.

Examples of question types used in Promise EBF

To enter the answer to a question, the user has to press the EDIT button. Based on the predefined input type of that question, a data type-specific input controller will be displayed to capture the data, figure 6. Commonly used question types are: Text answers, numeric answers, tick-off answers where either only one tick is possible (radio buttons) or more answers are possible (check boxes). In addition there is a question type where you have to answer "yes" or "no", and to distinguish between "no" and "not answered". There is also the possibility of specifying answers, e.g. when one uses an option like "other, please specify".

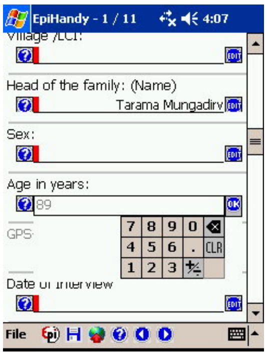

Figure 6a. Numeric input: A small calculator is used for number input where the user can only enter numbers.

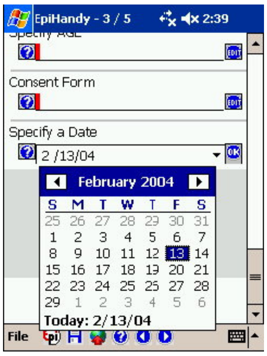

Figure 6b. Date: A typical calendar is used for easy selection of dates.

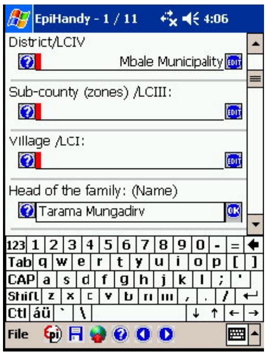

Figure 6c. Text input: There are 2 different question types for text entry: these are single line and multi-line (essay) as shown below, in combination with the onscreen keyboard text entry method.

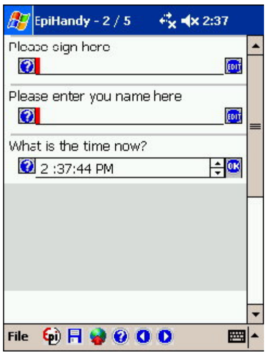

Figure 6d. Time: The time-function is mainly to record the actual time at the interview situation. If the question asks you to record another time, it can be entered by activating the automatic time and adjusting it.

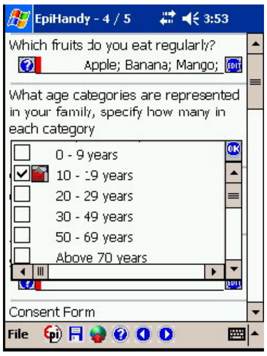

Figure 6e. Specify types: Specify types are created to add extra information related to a selected item in a list, i.e. specify types allow for responses that are more informative, e.g. if the list does not capture the informant's answer.

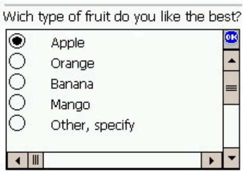

Figure 6f. List of items: Selection of items from a list can be of 2 types, exclusive select or multi-select; these are often referred to as radio buttons and check boxes. Radio buttons are based on exclusive selection, meaning that if one item is selected, this disables any other selected items.

Equipment and technical details

Server: The hardware equipment used at both sites was a regular computer/laptop that functioning as a server due to the low requirements of the EpiHandy Server software. Backup power with a UPS and voltage stabilizer was used to prevent data corruption due to power failures and fluctuations. Several laptops within the study office were also used to connect to the EpiHandy server using the EpiHandy StudyManager software.

GPS – Global Positioning System: In the Promise EBF study we used GPS location points for mapping and field verification of the origin of data. EpiHandy has this functionality built in by automatic reading from a connected GPS (cable or Bluetooth) or manual entry from a separate standalone GPS device. Due primarily to battery capacity issues, we used an external GPS connected to the handheld computer through a physical cable through the serial ports of the device. A challenge in using GPS is that one needs to be outside to get an accurate reading on the GPS. This was solved by the data collector – either before or after the interview – going outside and waiting until EpiHandy had captured a proper GPS signal before saving the document.

Handheld computer: Each of the data collectors had a handheld computer, HP iPAQ PocketPC with Windows Mobile 2003, each with a spare battery. Both batteries were charged every night. Each of the handheld computers was installed with EpiHandy MobileClient, had a separate GPS (Etrex Yellow) connected with a cable, and stored the data on a non-volatile memory card (Secure Digital – SD card). The batteries of the Etrex were regular rechargeable AA-sized batteries, which were charged about once a week in a separate charger. Figure 7 shows the main hardware components of the devices used for EpiHandy MobileClient in the Promise EBF study. Figures 8-14 shows the use of EpiHandy.

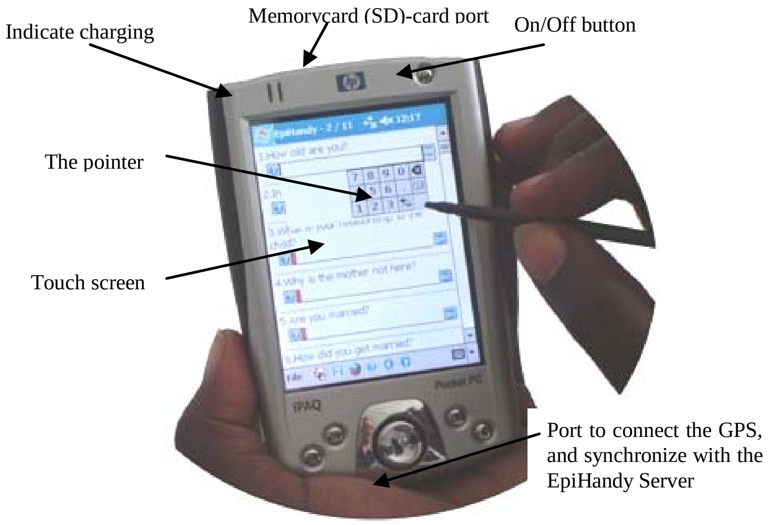

Figure 7. Handheld computer front view.

Implementation in Uganda

Training: The data collectors were trained on the interview tools; i.e. forms and procedures, before being trained on EpiHandy and data-capture with the handheld computer. The EpiHandy training consisted of a mix of step-by-step guided group presentations using a live display from the trainers handheld computer onto a projector screen, with hands-on simulations. As the forms were already familiar to the new users, the focus of the training was on the differences, showing the key functions, and troubleshooting, e.g. changing the battery while in the field. As part of the training of the questionnaires, the data collectors had to interview each other, perform role play and practice this as part of their homework (doing it in front of a mirror, trying to look up and not only down in the paper). After the initial training, data collectors were retrained 6 months later, at the beginning of the main study.

Data entry: Handheld computers were used to collect data using the EpiHandy software. Data collected on handheld computers were synchronized daily with a site server. The data manager made backups, kept track of interviews, and ensured that equipment and software functioned well. On a few occasions, data were initially collected on paper when the software was not functioning and/or due to prolonged periods of power failure. However, these questionnaires were entered into the EpiHandy system at the earliest convenience, normally by the following morning. Paper questionnaires were always carried by the data collectors as back-ups, but were seldom used. Some forms that were used only occasionally, e.g. verbal autopsies, missed visits, terminations, were only paper-based.

Data handling: The data manager ensured that all devices were properly charged and working before the data collectors signed out their respective device at the study office each morning. During the day, the field workers would collect data without needing any network connection. When they came back from the field with filled questionnaires on their handheld computer, they handed the devices over to the data manager, who connected the devices to the docking stations and uploaded data using the built-in synchronization features of EpiHandy. Any problems experienced in locating a household, completing a questionnaire, or conducting an interview were reported to the site coordinator.

Implementation in Burkina Faso

In Burkina Faso, the forms were translated into French using the built-in framework for multiple language support in EpiHandy. The study site was in a remote area and the field workers primarily resided in their respective data-capture areas; therefore they could not come to the study office every evening. There was neither electricity nor network coverage. The solution was to use 12-volt solar-charged car batteries and corresponding 12-volt chargers for the handheld computers. Data synchronization (from handheld computer to server) was problematic due to the remote locality. The solution to this problem was to buy additional SD (Secure Digital) memory cards for the handhelds. The memory cards where swappable; data could be shipped from the remote location to the data center by physically sending the memory cards one-by-one using existing delivery options. After arrival in the data center, the memory cards were inserted into a handheld computer similar to the one used in the field and the data offloaded from the memory card to the server.

Training and supervision of data collectors: The data collectors were given a 5-day training workshop, and a 2-day for field test of both paper-based questionnaires and the electronic data collection tools. Throughout the study, they received 3 refresher sessions and more than 18 supervisory rounds, including direct interviews, assessment interviews and data cross-checking. Data management was performed by an experienced data manager located in the data management center at Centre MURAZ, Banfora. Training and follow-up of data capture was done in conjunction with field supervisions and the monthly data collector meeting held each month in the Promise headquarters in Banfora.

EpiHandy in use

The following images show the EpiHandy in use in field settings for the Promise EBF study.

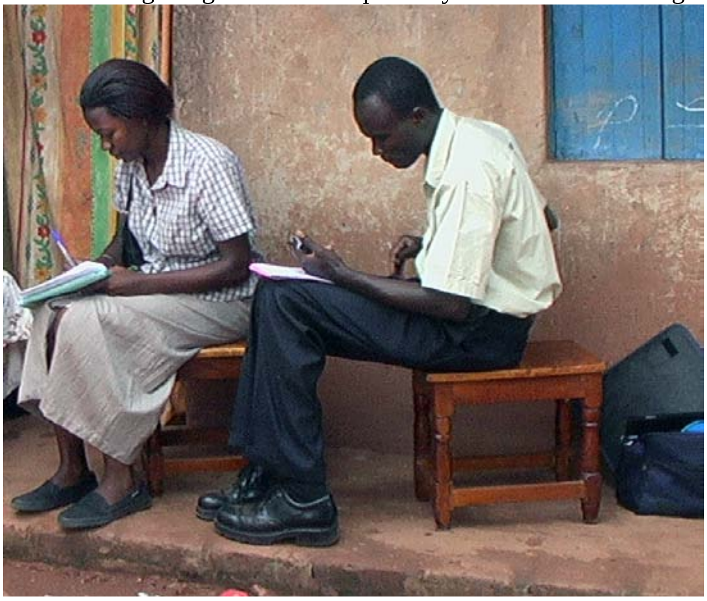

Figure 8. Fieldworkers conducting double entry, paper and electronic on a handheld computer during first field testing.

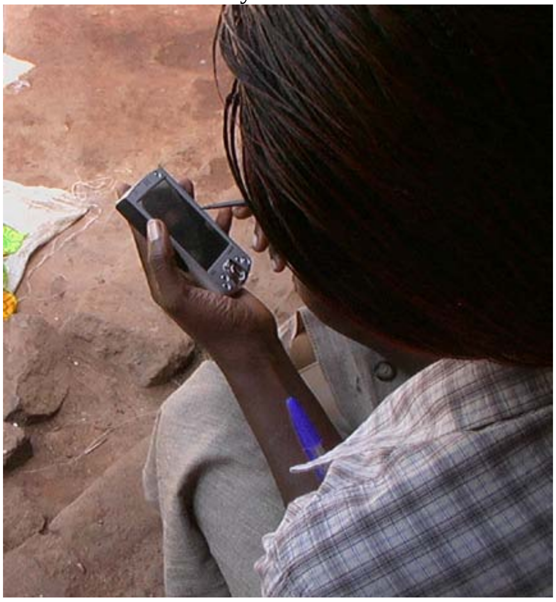

Figure 9. A fieldworker entering data about a child directly onto a handheld computer.

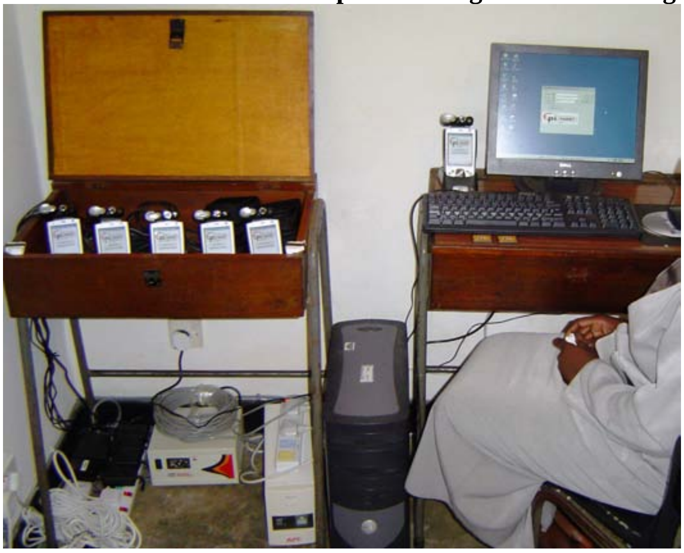

Figure 10. Initial setup of the EpiHandy server in the office where synchronisation with the handhelds takes place.

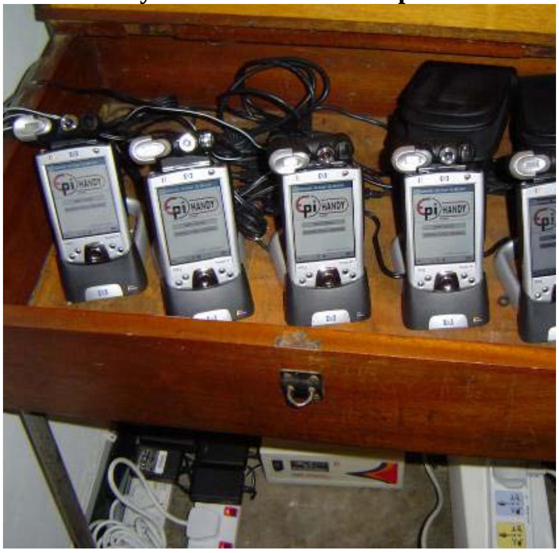

Figure 11. Handheld computers with EpiHandy in their charging cradles.

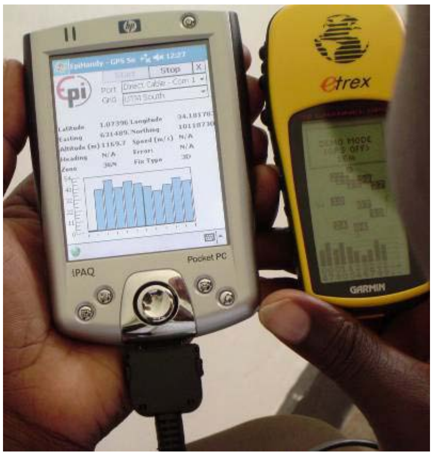

Figure 12. EpiHandy connected to GPS (Global Positioning System).

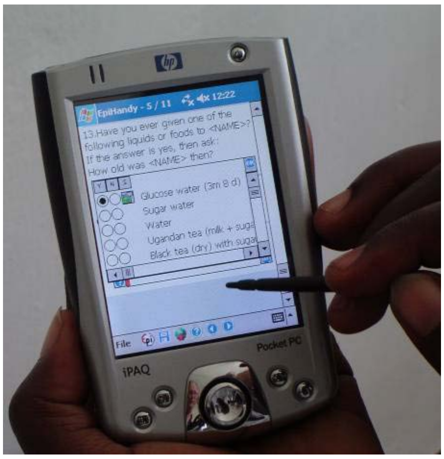

Figure 13. EpiHandy data-capture, showing part of a form.

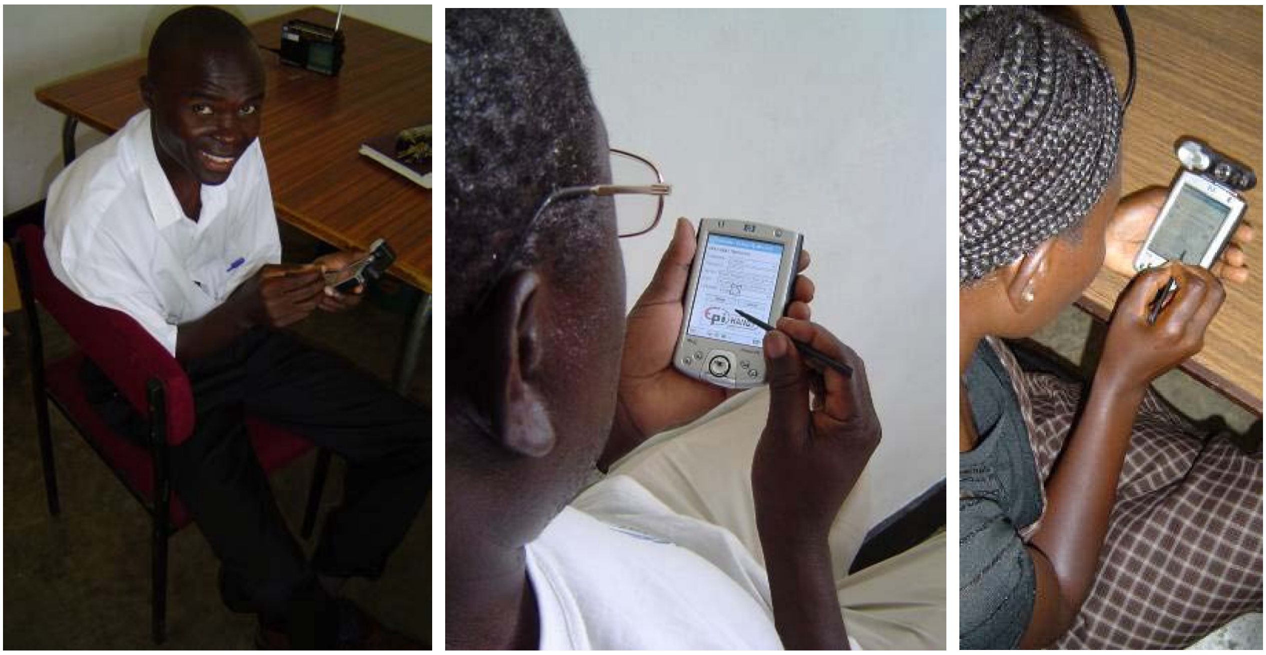

Figure 14. Fieldworkers testing data entry with EpiHandy on handheld computers.

J. Supplementary analysis

The analysis presented in the paper used all the included mother-infant pairs in the denominator, regardless of intervention intensity and participation in peer-counselling. This implies that we recoded missing, lost to follow-up and death as non-events, i.e. not having exclusively breastfed or not having had diarrhoea. We also undertook an alternative analysis excluding individuals lacking valid information, sometimes called ‘complete subject analysis’ (Tables 3-4). Only subjects with timely visits are included in the denominator in this analysis. The 2 analyses gave similar results.

Table 3. Exclusive breastfeeding (EBF) prevalence at 12 and 24 weeks of infant age, based on 24-hour recall and 7-day recall, and adjusted for clustering and site, and including only subjects with a timely visit in the denominator.

**12 weeks of infant age**						
	**Intervention**		**Control**			
	**N**	**(%)**	**n**	**(%)**	**PR***	**95% CI**
**Burkina Faso**						
EBF 24 hour recall	310/360	(86.1)	139/368	(37.8)	2.28	1.35-3.84
EBF 7 day recall	300/360	(83.3)	94/368	(25.5)	3.26	2.16-4.94
**Uganda**						
EBF 24 hour recall	323/369	(87.5)	161/323	(49.9)	1.77	1.60-1.96
EBF 7 day recall	305/369	(82.7)	125/323	(29.6)	2.15	1.87-2.48
**South Africa**						
EBF 24 hour recall	56/443	(12.6)	30/400	(7.5)	1.72	1.12-2.64
EBF 7 day recall	41/443	(9.3)	19/400	(4.8)	1.98	1.32-2.98

**24 weeks of infant age**						
	**Intervention**		**Control**			
	**N**	**(%)**	**N**	**(%)**	**PR***	**95% CI**
**Burkina Faso**						
EBF 24 hour recall	286/356	(80.3)	88/368	(23.9)	3.36	1.77-6.36
EBF 7 day recall	279/356	(78.4)	38/368	(10.3)	7.59	4.49-12.81
**Uganda**						
EBF 24 hour recall	232/360	(64.4)	57/321	(17.8)	3.68	2.86-4.73
EBF 7 day recall	203/360	(56.4)	41/321	(12.8)	4.47	3.20-6.25
**South Africa**						
EBF 24 hour recall	12/377	(3.2)	2/326	(0.6)	5.39	1.26-23.07
EBF 7 day recall	10/377	(2.7)	1/326	(0.3)	9.29	1.33-65.04

Adjusted for clustering and site

Table 4. Diarrhoeal morbidity by 2 week recall at 12 and 24 weeks of infant age and including only subjects with a timely visit in the denominator.

	Intervention		Control			
	n	(%)	n	(%)	PR*	95% CI
**12 weeks of age**						
Burkina Faso	20/360	(5.6)	36/368	(9.8)	0.57	0.25-1.22
Uganda	39/369	(10.6)	32/321	(10.0)	1.06	0.75-1.50
South Africa	45/443	(10.2)	33/400	(8.25)	1.12	0.77-1.63
**24 weeks of age**						
Burkina Faso	26/356	(7.3)	32/368	(8.7)	0.78	0.43-1.42
Uganda	52/360	(14.4)	59/321	(18.4)	0.78	0.54-1.12
South Africa	54/377	(14.3)	33/326	(10.1)	1.24	0.81-1.90

Intracluster correlation coefficients (ICC)

Intracluster correlation coefficients (ICC) for the 2 outcomes in the randomized clusters in Burkina Faso (24 clusters), Uganda (24) and South Africa (34), were very different (Table 5).

Table 5. Intracluster correlation coefficients (ICC) for reported exclusive breastfeeding and occurrence of diarrhoea at 2 time points, over the last two weeks in the randomized clusters in each of the countries.

	Burkina Faso	Uganda	South Africa
12 weeks of infant age			
EBF 24 hour recall	0.44521	0.11247	0.00000
EBF 7 day recall	0.40346	0.14951	0.00000
24 weeks of infant age			
EBF 24 hour recall	0.46396	0.19481	0.00226
EBF 7 day recall	0.52548	0.17108	0.00057
12 weeks of infant age			
Diarrhoea 2 week recall	0.03219	0.00000	0.02893
24 weeks of infant age			
Diarrhoea 2 week recall	0.02093	0.00617	0.04679

K. References

1. UNICEF. State of the world's children 2011, UNICEF, New York, 2010.
2. Hahn S, Puffer S, Torgerson DJ, Watson J. Methodological bias in cluster randomised trials. *BMC Med Res Methodol.* 2005;**5**:10.
3. Smith PG, Morrow RH. Field trials of health interventions in developing countries: a toolbox. 2nd ed. London: Macmillan Education; 1996.
4. Filmer D, Pritchett LH. Estimating wealth effects without expenditure data - or tears: an application to educational enrollments in states of India. *Demography* 2001;**38**:115-32.
5. Fadnes L, Taube A, Tylleskär T. How to identify information bias due to self-reporting in epidemiological research. The Internet Journal of Epidemiology. 2009;7:2

L. List of publications from the PROMISE EBF study to date

1. Daniels K, Nor B, Jackson D, Ekstrom EC, Doherty T. Supervision of community peer counsellors for infant feeding in South Africa: an exploratory qualitative study. *Hum Resour Health*. 2010; **8**: 6.
2. Diallo AH, Meda N, Zabsonre E, Sommerfelt H, Cousens S, Tylleskar T. Perinatal mortality in rural Burkina Faso: a prospective community-based cohort study. *BMC Pregnancy Childbirth*. 2010; **10**: 45.
3. Diallo AH, Meda N, Ouedraogo WT, Cousens S, Tylleskar T. A prospective study on neonatal mortality and its predictors in a rural area in Burkina Faso: Can MDG-4 be met by 2015? *J Perinatol*. 2011 Mar 3 [Epub ahead of print].
4. Engebretsen IM, Wamani H, Karamagi C, Semiyaga N, Tumwine J, Tylleskar T. Low adherence to exclusive breastfeeding in Eastern Uganda: a community-based cross-sectional study comparing dietary recall since birth with 24-hour recall. *BMC Pediatr*. 2007; **7**: 10.
5. Engebretsen IM, Tylleskar T, Wamani H, Karamagi C, Tumwine JK. Determinants of infant growth in Eastern Uganda: a community-based cross-sectional study. *BMC Public Health*. 2008; **8**: 418.
6. Engebretsen IM, Moland KM, Nankunda J, Karamagi CA, Tylleskar T, Tumwine JK. Gendered perceptions on infant feeding in Eastern Uganda: continued need for exclusive breastfeeding support. *Int Breastfeed J*. 2010; **5**: 13.
7. Engebretsen IM, Shanmugam R, Sommerfelt AE, Tumwine JK, Tylleskar T. Infant feeding modalities addressed in two different ways in Eastern Uganda. *Int Breastfeed J*. 2010; **5**: 2.
8. Fadnes LT, Engebretsen IM, Wamani H, Semiyaga NB, Tylleskar T, Tumwine JK. Infant feeding among HIV-positive mothers and the general population mothers: comparison of two cross-sectional surveys in Eastern Uganda. *BMC Public Health*. 2009; **9**: 124.
9. Fadnes LT, Engebretsen IM, Wamani H, Wangisi J, Tumwine JK, Tylleskar T. Need to optimise infant feeding counselling: a cross-sectional survey among HIV-positive mothers in Eastern Uganda. *BMC Pediatr*. 2009; **9**: 2.
10. Fadnes LT, Engebretsen IM, Moland KM, Nankunda J, Tumwine JK, Tylleskar T. Infant feeding counselling in Uganda in a changing environment with focus on the general population and HIV-positive mothers - a mixed method approach. *BMC Health Serv Res*. 2010; **10**: 260.
11. Fadnes LT, Nankabirwa V, Sommerfelt H, Tylleskar T, Tumwine JK, Engebretsen IM. Is vaccination coverage a good indicator of age-appropriate vaccination? A prospective study from Uganda. *Vaccine*. 2011; **29**: 3564-70.
12. Fadnes LT, Jackson D, Engebretsen IM, Zembe W, Sanders D, Sommerfelt H, Tylleskar T, Promise-EBF Study Group. Vaccination coverage and timeliness in three South African areas: a prospective study. *BMC Public Health.* 2011; **11**: 404.
13. Fjeld E, Siziya S, Katepa-Bwalya M, Kankasa C, Moland KM, Tylleskar T. 'No sister, the breast alone is not enough for my baby' a qualitative assessment of potentials and barriers in the promotion of exclusive breastfeeding in southern Zambia. *Int Breastfeed J*. 2008; **3**: 26.
14. Nankabirwa V, Tylleskar T, Tumwine JK, Sommerfelt H. Maternal education is associated with vaccination status of infants less than 6 months in Eastern Uganda: a cohort study. *BMC Pediatr*. 2010; **10**: 92.
15. Nankabirwa V, Tumwine JK, Tylleskär T, Nankunda J, Sommerfelt H. Perinatal mortality in Eastern Uganda: a community based prospective cohort study. *PLoS ONE*. 2011;**6**:e19674.
16. Nankunda J, Tumwine JK, Soltvedt A, Semiyaga N, Ndeezi G, Tylleskar T. Community based peer counsellors for support of exclusive breastfeeding: experiences from rural Uganda. *Int Breastfeed J*. 2006; **1**: 19.
17. Nankunda J, Tumwine JK, Nankabirwa V, Tylleskar T. "She would sit with me": mothers' experiences of individual peer support for exclusive breastfeeding in Uganda. *Int Breastfeed J*. 2010; **5**: 16.
18. Nankunda J, Tylleskar T, Ndeezi G, Semiyaga N, Tumwine JK. Establishing individual peer counselling for exclusive breastfeeding in Uganda: implications for scaling-up. *Matern Child Nutr*. 2010; **6**: 53-66.
19. Nkonki LL, Daniels KL. Selling a service: experiences of peer supporters while promoting exclusive infant feeding in three sites in South Africa. *Int Breastfeed J*. 2010; **5**: 17.
20. Nor B, Zembe Y, Daniels K, Doherty T, Jackson D, Ahlberg BM, et al. "Peer but not peer": considering the context of infant feeding peer counseling in a high HIV prevalence area. *J Hum Lact*. 2009; **25**: 427-34.
21. Wandera M, Engebretsen IM, Okullo I, Tumwine JK, Astrom AN. Socio-demographic factors related to periodontal status and tooth loss of pregnant women in Mbale district, Uganda. *BMC Oral Health*. 2009; **9**: 18.
22. Wandera MN, Engebretsen IM, Rwenyonyi CM, Tumwine J, Astrom AN. Periodontal status, tooth loss and self-reported periodontal problems effects on oral impacts on daily performances, OIDP, in pregnant women in Uganda: a cross-sectional study. *Health Qual Life Outcomes*. 2009; **7**: 89.
