# Supplementary figures and images for: Effect of Breastfeeding Promotion on Early Childhood Caries and Breastfeeding Duration among 5 Year Old Children in Eastern Uganda: A Cluster Randomized Trial
Source: PLoS One. 2015 May 4;10(5):e0125352. doi: 10.1371/journal.pone.0125352 (PMC4418833; doi:10.1371/journal.pone.0125352)

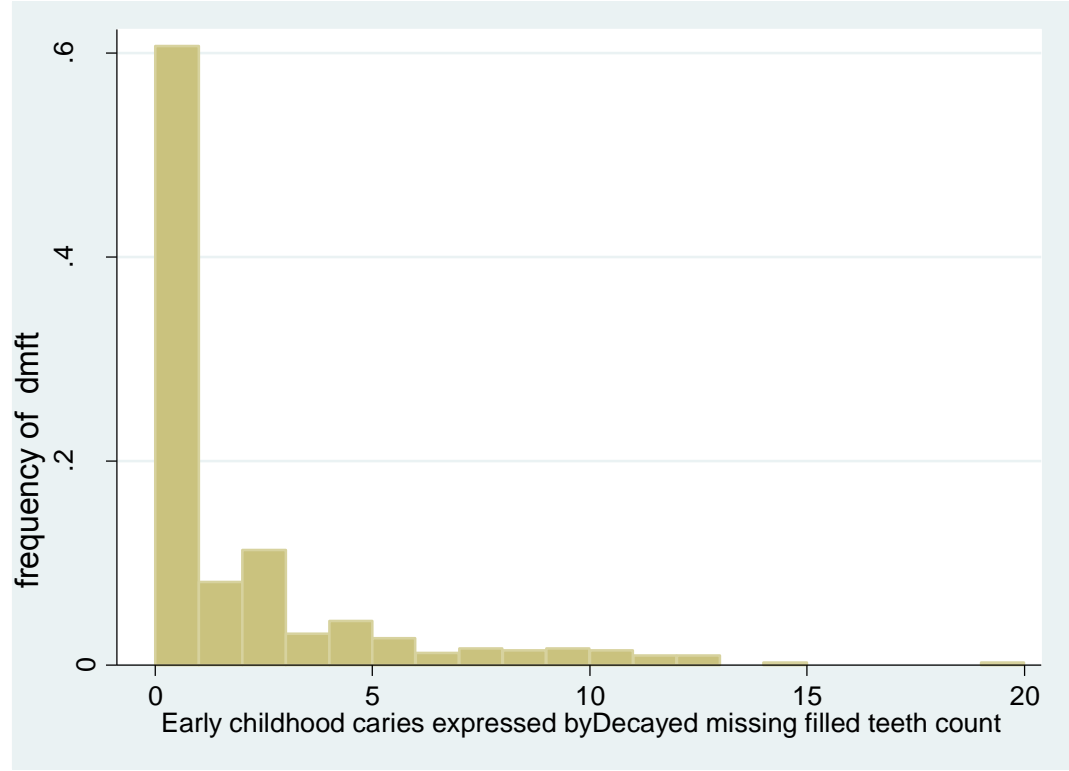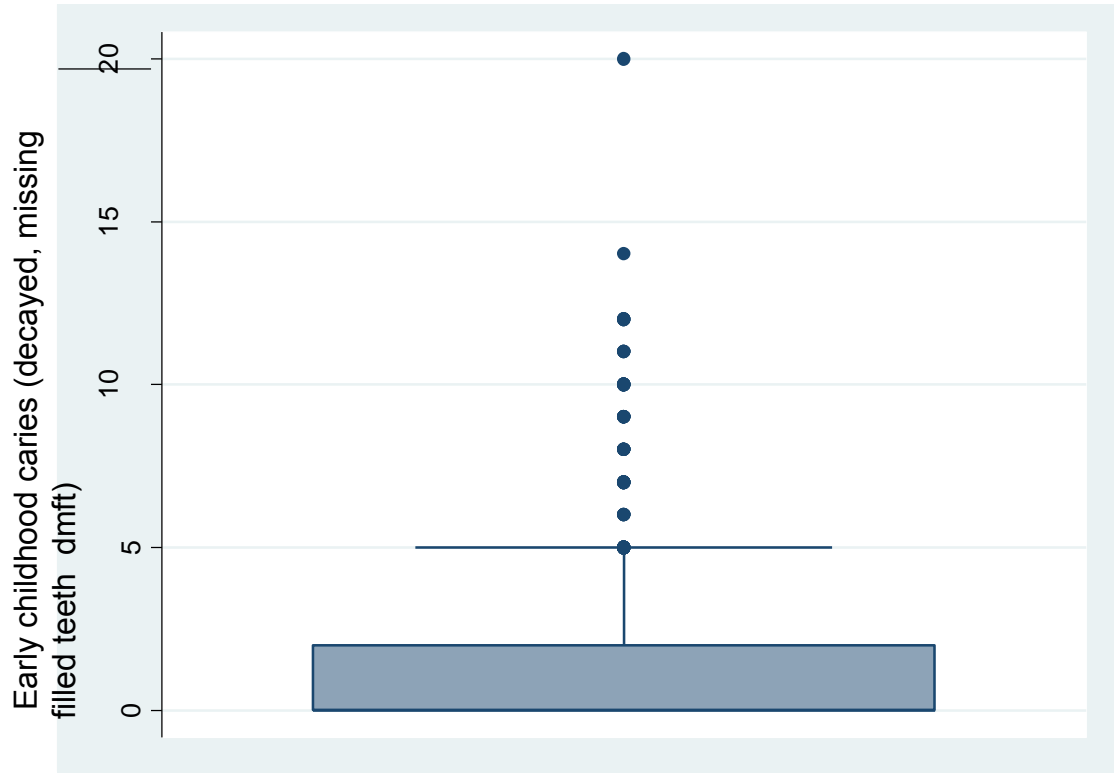

Supplement: S2 File — (PDF) [file pone.0125352.s003.pdf]
