# Supplementary material for: Effect of Breastfeeding Promotion on Early Childhood Caries and Breastfeeding Duration among 5 Year Old Children in Eastern Uganda: A Cluster Randomized Trial
Source: PLoS One. 2015 May 4;10(5):e0125352. doi: 10.1371/journal.pone.0125352 (PMC4418833; doi:10.1371/journal.pone.0125352)
Supplement: S1 Protocol — (PDF) [file pone.0125352.s004.pdf]

Title:

Multicentre, community-randomised, controlled trial comparing the safety and efficacy of peer-support for exclusive breastfeeding compared to standard care.

**Protocol no.:**	INCO-CT-2004-003660
**Trial co-ordinator:**	University of Bergen
**Principle Investigators:**	Professor Thorkild Tylleskar<br>Professor Philippe Vande Perre<br>Dr Lotta Ekström<br>Dr Nicolas Meda<br>Professor James Tumwine<br>Dr Chipepo Kankasa<br>Dr Mickey Chopra
**At sites:**	Centre Muraz: Dr Nicolas Meda<br>Makerere University: Professor James Tumwine<br>University of Zambia: Dr Chipepo Kankasa<br>University of Western Cape: Dr Mickey Chopra
**Sponsor:**	Investigator Driven Trial
**Protocol date:**	30/03/2006
**Revised:**	**07/12/2007**
**Protocol version:**	**One – Amendment 3**

Full title

PROMISE EBF: Promoting Infant Health and Nutrition in Sub-Saharan Africa: Safety and Efficacy of Exclusive Breastfeeding Promotion in the Era of HIV: a multicentre, community-randomised, controlled trial comparing the safety and efficacy of peer-support for exclusive breastfeeding compared to standard care.

Short title

PROMISE EBF: Safety and Efficacy of Exclusive Breastfeeding Promotion in the Era of HIV in Sub-Saharan Africa

Table of contents **Page number**

1.0 Introduction 4
1.1 Exclusive breastfeeding – an even better version of breastfeeding 4
1.2 Breastfeeding and risk of HIV transmission 5
1.3 EBF and micronutrient status 6
1.4 The intervention: Peer Counselling 6
1.5 Potential impact 7
2.0 Objectives 9
2.1 Primary objectives 9
2.2 Secondary objectives 9
3.0 Methodology 10
3.1 Design 10
3.2 Study settings 10
3.3 Target population 10
3.4 Inclusion criteria 10
3.5 Exclusion criteria 10
3.6 Randomization 11
3.7 Sample size and power 11
3.8 Recruitment procedure 12
3.9 Participant flow 12
3.10 Intervention and standard care 13
3.11 Primary outcome 13
3.12 Secondary outcomes 13
3.13 Data collection and data capture 13
3.14 Validity and reliability 15
3.15 Data analyses 15
4.0 Economic evaluation 15
5.0 Ethical considerations 16
6.0 Plan for using and disseminating knowledge 17
6.1 Management of knowledge and intellectual properties 18
6.2 Publications 18
7.0 Potential conflict of interest 18
8.0 Project management structure 18
8.1 The coordinator 18
8.2 Steering Committee 19
9. Logical time framework 20
10.0 References 21
11.0 Appendices 23

PROMISE EBF: Promoting Infant Health and Nutrition in Sub-Saharan Africa: Safety and Efficacy of Exclusive Breastfeeding Promotion in the Era of HIV: a multicentre, community-randomised, controlled trial comparing the safety and efficacy of peer-support for exclusive breastfeeding compared to standard care.

1.0 Introduction

The single most effective way of saving the lives of millions of young children in developing countries would be the promotion of exclusive breastfeeding (EBF). Over a period of 10 years it could save the lives of an estimated 15 million children.[1]

> *"Infants aged 0-5 months who are not breastfed have seven-fold and five-fold increased risks of death from diarrhoea and pneumonia respectively, compared with infants who are exclusively breastfed. At the same age, non-exclusive rather than exclusive breastfeeding results in more than two-fold increased risks of dying from diarrhoea and pneumonia."*[2]

This assertion, and related comments and findings, set out by the Bellagio Child Survival Group in a recent article in the Lancet summarising findings from international research of how best to reduce infant mortality in developing countries, is made despite our limited knowledge of how best to promote EBF. This proposal addresses the urgent need to tackle this limitation. How best to promote EBF needs attention because it will be necessary to secure high rates of support for EBF from young mothers, and we do not currently know the best way to bring this about, particularly in the cultural context of Africa. Unless we can find more effective strategies to combat poor feeding practices in both health facility and community settings, the child mortality will continue to be high.

The benefits of breastfeeding and the negative effects of artificial feeding in underprivileged environments were clearly brought to public attention in the 1970'ies, backed by increasingly strong scientific support. As a result the International Code of Marketing of Breast-milk Substitutes and subsequent World Health Assembly Resolutions were created in the 1980'ies to avoid marketing of infant foods, teats and bottles in ways that could interfere with breastfeeding. In the early 1990'ies, UNICEF started the "Baby-friendly Hospital Initiative" (BFHI), which decreased many of the practices in the health care system that had a negative impact on breastfeeding. Together with many of the other child health programmes during these decades these interventions contributed to a reduction in child morbidity and mortality.

1.1 Exclusive breastfeeding – an even better version of breastfeeding

EBF means that the infant receives nothing else than breast milk, not even water. That breastfed

children do not need anything besides breast milk, not even water, and not even in hot climates, was discovered in the 1970s.[3] A decade of research confirmed this, followed by an attempt to define breastfeeding into various patterns by WHO[4] and others.[5] Since then, research has begun to suggest that, even in developed countries, EBF protects against gastrointestinal, respiratory and other infections, and that the protective effect is enhanced with greater exclusivity of the breastfeeding.[6] Exclusivity also increases the duration of amenorrhoea, positive for maternal health and birth spacing.[6]

EBF is not a traditional concept; beliefs and practices regarding a wide range of early supplements are common around the world, and often seem to reflect beliefs expressed by early physicians such as Galen.[7] Nor is it widely understood by health care professionals, given the lack of information on it even in most paediatric basic training.

Thus EBF is not widely practised anywhere except perhaps in much of Scandinavia and parts of some countries in other regions, including a few African countries. The best source of data on the duration of exclusive breastfeeding in developing countries is the Demographic and Health Surveys run by Macro International Inc. and funded by USAID. In only 23% of the 61 national surveys from Sub-Saharan Africa available online: (http://www.measuredhs.com/statcompiler/table_builder.cfm?userid=90787&usertabid=101224) is the median duration of exclusive breastfeeding longer than one month. Even this overestimates the amount of exclusive breastfeeding being done in Africa, as the indicator used is not “EBF since birth,” but “EBF the day before the survey.”

So if EBF is able to lower child mortality by 13%, why is it not promoted on a large scale already today? Two major reasons are: fear of HIV transmission through breast milk and uncertainty about the micronutrient status in children exclusively breastfed for 6 months.

1.2 Breastfeeding and risk of HIV transmission

When an HIV-positive mother breastfeeds for 1.5 years or more, this appears to lead to an additional 15% risk of postnatal HIV transmission on average. At a population level the epidemiological evidence is that exclusive breastfeeding should still be promoted even in countries with high prevalence of HIV.[8] For many HIV positive mothers the risk of transmitting HIV to their children is outweighed by the risk of the infant dying if not breastfed because of the poor socio-economic and environmental conditions they live in.[9] Limited evidence suggests that for these women and their children EBF is even more important, not just for minimising respiratory and gastrointestinal infections but also perhaps in reducing the risk of HIV transmission.[10] However in reality the HIV epidemic has undermined breastfeeding promotion efforts especially in countries that would benefit the greatest.[11] In addition, expensive approaches of dealing with HIV

have removed the focus from breastfeeding as an inexpensive way of promoting optimal child health.[1]

While the exact mechanism for HIV transmission during breastfeeding is unknown, there are a range of known maternal risk factors including nutritional status,[12] viral loads in plasma and breast milk[13] and CD4 count,[14] breast pathologies,[13] including subclinical mastitis[15, 16]. The longer the breastfeeding lasts, the greater the transmission risk.[17, 18] Oral thrush in the infant may also increase risk of transmission.[19] One study in which infants were exclusively breast-fed for 3 months found that the risk of transmission appeared to be greatly reduced compared with the normative pattern of mixed feeding.[10] On the other hand non-breastfeeding also has a detrimental effect on child survival. Breastfeeding still seems to be a reasonable trade-off for poor African HIV-positive mothers, particularly if exclusive.

Piwoz and Ross[20] estimate that perhaps half of all breastfeeding transmission of HIV may be due to the breast problems mentioned above. Thus lactation management needs to be readily available to assist women to breastfeed properly from the outset and to reduce breast damage when problems do arise.

1.3 EBF and micronutrient status

Exclusively breast-fed babies appear to grow well for up to six months in both developed and developing countries,[21] suggesting that severe deficiency of most micronutrients is unlikely. However, in recently deciding to recommend EBF for six months,[22] WHO warned that, based on one randomised trial in Honduras,[23] infant iron status might not be optimal at six months and that data were inadequate to judge what effect six months of exclusive breastfeeding might have on infant status regarding certain other micronutrients, particularly zinc and vitamin A.[24-26] That such basic research to establish the “normal” micronutrient status of infants exclusive breast-fed from birth has not been done reflects the fact that almost no researchers have utilised a strict definition of EBF and that very few infants in most countries have been fed that way. The use of a strict definition in doing such research would seem justified, as the gut environment, including its microflora, is likely to influence the absorption of certain micronutrient but also to be influenced by the addition of most types of supplements and even medications such as antibiotics.

1.4 The intervention: Peer Counselling

The constraints discussed above have meant that efforts to promote EBF have been rudimentary so far, consisting mainly of the BFHI. But in Africa nearly all such efforts have come almost to a complete standstill in recent years. EBF promotion therefore needs to be reshaped in this era of HIV. The challenge is urgently to put back on track the promotion of exclusive breastfeeding for

the benefit of the world's children without losing credibility by doing it indiscriminately. PROMISE will to fine-tune and test an upgraded version of EBF-promotion that is HIV-sensitive and to do it in four African countries with a high prevalence of HIV where it is most needed. In particular, it will be the first study to deal explicitly with each of these constraints, opening the way for more widespread and successful promotion of EBF, especially in Africa, ultimately giving back to Africa one of the powerful and cheap interventions to prevent child mortality and morbidity.

Peer counselling is a proven cost-effective approach for changing behaviour, for example, preventing HIV infection[27]. Several studies have also examined its impact on breastfeeding behaviour. Based largely on quasi-experimental studies peer counselling was shown to have an impact on the duration of any breastfeeding.[28-32] Kristin et al in the USA[33] obtained increases in EBF among women who obtained services from peer counsellors compared to those for whom requested services were not available; Haider et al[34] in Bangladesh in a trial randomised by community and Morrow et al[35] in a trial randomised at individual level in Mexico also obtained substantial increases. However, in the USA two trials failed to have much impact.[36, 37] Green[38] summarised the results of four unpublished quasi-experimental studies. Impacts of peer counselling in intervention compared to comparison/control groups were seen in studies done in Chile and Honduras. In another study in Honduras and one in Mexico, little effect was achieved, presumably due to limited implementation. Thus we feel that peer counselling is promising enough to be chosen as the intervention to focus on but not proven clearly enough (indeed, hardly tested at all in Africa) to prevent it from being ethically randomised.

1.5 Potential impact

This study will provide the first data from a randomised trial on the safety and efficacy of peer counselling as a way of increasing rates of exclusive breastfeeding in the African context. Outcomes include morbidity, growth, and micronutrient status.

The range of four countries included will enhance generalisability of these findings. The study will provide measures of the impact of this intervention on infant morbidity, growth and micronutrient status as well as the costs, efficacy and implications for the health care system of implementing such an intervention. Thus it will in one package provide all the information policy makers need and hopefully will thus lead to more rapid replication if the outcome is positive. Peer counsellors are by definition local women and thus a low-cost intervention suitable to resource-poor environments.

In Europe the health implications of exclusive breastfeeding for six months are not well known, perhaps largely because it has been rare. A relatively long period of EBF has however been the norm in Norway and Sweden for over a decade, and randomised trials comparing various

outcomes for 4 vs. 6 months EBF are ongoing in Sweden and Iceland. Four months of EBF is rare in most of Europe (WHO Global Data Bank on Breastfeeding http://www.who.int/nut/db_bfd.htm) and yet widely agreed upon to be important for infant health even in Europe.[39-43] As far as we know, efforts to promote it in Europe have been limited. Thus it is important for European universities to learn how this can be done.

PROMISE will generate cross-fertilization among the three European Universities to learn from and share with each other and the African colleagues several areas of research. None of the partners are experts in all these areas (breastfeeding promotion, micronutrients, HIV). We expect a long-term research consortium to emerge from this beginning.

PROMISE EBF will provide an example to the world of how efforts to promote exclusive breastfeeding and avoid postnatal HIV transmission can be done jointly, and what synergisms may result. Methods employed and costs will be documented and reported on to increase ease of replication and sustainability.

In Africa, there is a growing awareness that strategies must be identified and tested that can lead to the highest possible infant survival in the face of the risk of postnatal HIV transmission. In 1997 UNAIDS, WHO and UNICEF changed their policy on HIV and infant feeding, and issued guidelines for implementing this new policy a year later. Whereas previously countries where most infant mortality was due to infection and malnutrition were advised to promote breastfeeding irrespective of the mother's HIV status, the new policy called for individual counselling on the advantages and disadvantages of breastfeeding vs. artificial feeding that would enable mothers to make an informed choice. UNICEF provided free formula at a number of pilot sites.

Later a World Health Assembly Resolution recommended that replacement feeding be advised where it was acceptable, feasible, affordable, safe and sustainable. In response to concerns that pilot site approaches are being expanded without taking into account the potential importance of exclusive breastfeeding, several UN agencies have endorsed a new "Framework for Priority Actions" for countries that want to go to scale with such approaches.[44]

2.0 Objectives

The objective of this study is to lead the way in the promotion of child health by developing, implementing and assessing the health impact of an intervention to promote exclusive breastfeeding in African settings where a high prevalence of HIV is a barrier.

2.1 Primary objectives

The primary objectives are to assess in a community-randomised trial with intention-to-treat analysis:

1. the impact of peer-counselling in increasing the exclusive breastfeeding rates at 3 months of age and
2. the effects of the trial on infant morbidity (2 weeks diarrhoeal disease point prevalence at 3 months)

2.2 Secondary objectives

The secondary objectives are:

1. To assess the effects of the trial on growth (up to 6 months of age) and micronutrient status
2. To relate infant morbidity, growth, micronutrient status to actual feeding pattern
3. To observe how micronutrient status develops in exclusively breast-fed infants up to six months of age
4. To assess the cost-effectiveness of the interventions in terms of cost per averted child morbidity, and per additional Disability Adjusted Life Year
5. To assess the data collection and handling for field trials using new handheld computer technology
6. To describe the quality, efficiency and equity dimensions of the interventions and to analyse how best to optimise the sustainability of the intervention for the health care sector
7. Transfer of technology and capacity regionally between the African countries, the European countries and between the continents
8. Training of young researchers at the seven partner institutions within their own institution but also through exchange among the partners
9. Building a research consortium that can sustain the collaboration developed in this study

3.0 Methodology

3.1 Design

A prospective, multi-centre, community-randomised, controlled, experimental design will be used to execute this trial.

3.2 Study settings

The study will be carried out in four African countries, Burkina Faso in French-speaking West Africa, Uganda in East Africa, Zambia in Central Africa and South Africa in Southern Africa.

In each country one of more districts have been selected as the intervention sites, Banfora in the South-west of Burkina-Faso, Mbale District in Eastern Uganda, Mazabuka in Southern Province in Zambia and three sites in South Africa: Umlazi in Durban, KwaZulu Natal, Rietvlei in Eastern Cape and Paarl in Western Cape. Pregnant women in the randomised communities (=clusters) will be informed about the study and asked if they are willing to participate. If they agree, an enrolment interview will be carried out and based on eligibility criteria the women will or will not be accepted into the study.

3.3 Target population

The target population will be pregnant mothers who opt to breastfeed their infants who conform to the inclusion and exclusion criteria.

3.4 Inclusion criteria

The inclusion criteria are:

- Lives in a selected cluster
- Is pregnant ≥ 6 months or having given birth less than 7 days ago
- Has no plans to move outside the cluster within 1 year

3.5 Exclusion criteria

The exclusion criteria are:

- Reduced ability to collaborate for psychological/mental reasons
- Severely ill
- Having given birth more than 7 days ago
- Having given birth less than 7 days ago and it was a multiple birth, or the child has a severe malformation, or death of the mother or the baby.

3.6 Randomization

A number of over 30 randomisation units should be identified within reasonable distance from the study office. The randomisation unit varies between sites but will typically be 1-2 villages with an average of 1000 inhabitants (about 35 infants born per year given a birth rate of 3%). Before engaging in the selection of communities, extensive information will be given to community leaders and the rest of the selection process will be done in close collaboration with community leaders. Within the district the suitable communities will be selected and stratified groups considered being similar, based on location, urban-rural, socio-economic status. Care will be taken to allow for 'corridors' between selected communities to be randomised. In each stratum half of the communities will be randomised to intervention and the reminder to control communities. Local community leaders will be further informed about the study.

Each of the clusters should be visited and checked for size, collaborative administrative leaders, not sharing important facilities (water supply, market/shop/trading centre, primary school, mosque, church) with any other potential cluster. If both urban and rural clusters are included no less than 30% of each should be included. The complete list of randomisable clusters will be checked and 24 clusters will be randomised in each site, if applicable in rural/urban strata.

3.7 Sample size and power

Of the primary outcome measures the decrease in diarrhoea is the most difficult to catch. We have based our sample size calculation on the expected reduction in cases of diarrhoea (at the age of 3 months) over the last two weeks of one third from 12% to 8%. With a 95% confidence (alpha error 0.05) and power of 80%, and an average number of infants of 35 per randomised community, and a coefficient of variation between the communities of 0.3, we will need to randomise 48 communities in each arm – a total of 96 communities, see table below.

		Increase in EBF from 20 to 40%	Decrease in diarrhoea
Proportion in the intervention group	P1	0.4	0.08
Proportion in the control group	P2	0.2	0.12
Percentage point for alfa error = 0.05	z1	1.96	1.96
Percentage point for beta error = 0.20	z2	1.28	1.28
Number of individuals in each community	n	35	35
coefficient of variation of proportions among communities in each group	k	0.4	0.3
Average of P1 and P2	P	0.3	0.1
Number of communities needed per arm	C	12	48

This will include 420 infants in each arm in each country, a total of 840 children per country and all in all 3360 children studied across the four sites. This sample size will very accurately give us the increase in EBF and be able to document the above decrease in diarrhoea morbidity.

3.8 Recruitment procedure

The recruitment procedure will be adapted to the local circumstances. In the most common case, each cluster will have a recruiter ("pregnancy monitor") who will be a mature woman of good reputation living within the cluster. The pregnancy monitors will inform the research centre about pregnant women that may be eligible to participate in the study. A data collector / research assistant will be sent to visit the woman to ascertain eligibility, obtain consent to participate in the study and conduct the recruitment interview.

In South Africa, the recruitment of women will be done at the health facility in the antenatal clinic, where a data collector / research assistant will interview the woman to ascertain eligibility, obtain consent to participate in the study and conduct the recruitment interview.

All mothers in the selected and randomised clusters are a priori selected and randomised. In order to carry out an intention-to-treat analysis, it is important to minimise the loss of eligible mothers. For mothers declining to participate, minimal socio-demographic information should preferably be collected.

3.9 Participant flow

Pregnant women will be identified by a recruiter ("pregnancy monitor")
The pregnancy monitors will inform the research centre about the pregnant woman
A data collector / research assistant will visit the woman to ascertain eligibility
Consent to participate in the study will be obtained
The recruitment interview will be conducted
The peer-counsellor is informed about the woman and visits her to make an appointment
The woman is visited by the peer-counsellor who provides the first counselling session
The woman gives birth
The woman is revisited by the peer-counsellor after birth who provides the 2nd counselling session
The woman is revisited by the peer-counsellor after birth who provides the 3rd counselling session
A data collector / research assistant will visit the woman for the week 3 interview
The woman is revisited by the peer-counsellor after birth who provides the 4th counselling session
A data collector / research assistant will visit the woman for the week 6 interview
The woman is revisited by the peer-counsellor after birth who provides the 5th counselling session

A data collector / research assistant will visit the woman for the week 12 interview
A data collector / research assistant will visit the woman for the week 24 interview

3.10 Intervention and standard care

The study will have two arms: An intervention arm and a standard care arm.

Intervention arm

The infants in the intervention arm will be treated similarly to the infants in the standard care arm except for the peer-counselling on exclusive breastfeeding. After the informed consent, the mothers in the intervention arm will be visited by a peer-counsellor on 1 occasion before delivery, at 0.5-1, 2, 4, 8 (and 16 weeks) postnatally. The peer-counsellor will deliver messages on the advantages of EBF; provide support for EBF, provide simple technical information for overcoming constraints, provide referral of more complicated problems, and provide reminders and encouragement to remain/return to EBF.

3.11 Primary outcome

The outcome measures in this study are:

- EBF prevalence at 3 months. In the intervention group receiving peer counselling, we expect an raise in the proportion of infants who are exclusively breast-fed from birth through 3 months by 20 percentage points from baseline of 20% (although we will recommend 6 months, we do not expect many to achieve this in any group). This measure will be site specific.
- The impact of increased EBF on infant morbidity, growth and micronutrient status requires a larger sample. We have decided to use active surveillance for infant morbidity and we use the following outcome measures, which will be calculated for the whole study:
- Decrease in diarrhoea. In the intervention group we expect a reduction of one third from an estimated baseline of 12 % diarrhoea incidence in the last 2 weeks (to 8% in the intervention group).

3.12 Secondary outcomes

- Improved growth up to 6 months of age.
- Micronutrient status. We have decided to assess infants in three of the sites, Burkina Faso, Uganda and Zambia. If no major deviation is seen in such a sample, we conclude there are no pertinent micronutrient deviations.

3.13 Data collection and data capture

- Data collection will be carried out using the software EpiHandy, a data collection tool for handheld computers. Questionnaires are designed on a desktop PC using EpiHandy -

SurveyManager, downloaded to handheld computers, displayed in a user-friendly form using local languages. The interviewers enter the data directly on the handheld computer by clicking on the touch sensitive screen. There is an instant check for validity, which does not allow certain types of erroneous responses to be entered. Using a handheld GPS connected to the handheld, the geographical position of each interview is automatically recorded. Collected data is temporarily stored in a secure and encrypted database on a memory card on the handheld and automatically uploaded to the site-specific database on a daily basis.

- A data management centre will be located in South Africa at an established research centre. The databases in each site will be maintained by local computer staff. The data from the sites will be transferred on weekly basis to the data management centre to minimise the risk of data loss. The transfer will be based on using established and secure technologies that will be setup for this purpose. From this centre anonymous datasets will be made available to the principal investigators for further analysis.
- Access to collected data at the site will be strictly controlled by the onsite data manager. This security will be possible through the use of industry standard SQL databases i.e. MS SQL Server or MSDE (Microsoft Desktop Engine) that have high level of reliability and built-in security. The data stored on the handheld computer will be encrypted and protected with a strong password and username combination. Site offices will be secured with appropriate measures to prevent theft and unwanted access to hardware and data. Strict backup procedures at site level and at the data management centre will be established, these will include daily backup to appropriate media which will be safely stored. The weekly transfer of data from sites to the data management centre will be an additional backup method.
- Local field staff will receive training on the use of handheld computers and the data collection software (EpiHandy), this will be done in combination with training on interview techniques and anthropometric measurements. The same training materials will be used across all sites to ensure standardisation. A data manager will be trained in management of the database, systems used and support to the interviewers using the handheld computer.
- Trained interviewers will visit and interview all the trial mothers at recruitment and at 3, 6, 12 and 24 weeks postnatally, to administer pretested questionnaires and perform anthropometric assessment of the child. Using handheld computers the data will be checked for consistency and validated at the time of entry.
- Standard operating procedures (SOP’s) will be developed for each stage of data collection, handling and analysis. These will include data validation, methods for data cleaning and quality control.

3.14 Validity and reliability

Inferential validity refers to the validity of the logical inferences (inductive and deductive) or conclusions that will be drawn from the results of this research project. Statistical tests of chance, confidence intervals and relative risk will be used to determine the differences between the outcomes of the two groups. Conclusions will be made, based on the differences between the outcomes in the two groups. External (universal) validity is concerned with the extent to which the results can be applied beyond the sample population. External validity will be achieved by using a detailed trial procedure, with specific inclusion and exclusion criteria as well as a calculated sample size adequate to give reliable results. A detailed trial procedure (protocol) will be used to ensure inter - researcher reliability for the execution of the project.

3.15 Data analyses

Appropriate statistical analysis will be performed by trained staff in close collaboration with the senior scientists in the project. A detailed SOP will be developed for this. The analysis will include an aspect of training and capacity building of young researchers.

4.0 Economic evaluation

The economic evaluation is divided in the development of costing tools and cost-effectiveness analysis.

At facility level, forms will be developed to register units of recurrent cost-items consumed, the labour consumption of different labour categories, and the alternative cost of these resources. The facility level form will also include questions to calculate the annualized cost of buildings and other capital items, and the share of the interventions in these resources. Training costs of staff will also be included in this form. At patient level, a questionnaire will be developed to assess the direct and indirect costs patients face utilising the interventions. At health facility level, resource use will be registered as part of daily routines by the regular staff. Alternative costs of the resources will be assessed by the research group. Specially trained enumerators will do structured interviews with patients to assess the patient’s costs. The data analysis will be done at aggregated and disaggregated levels using standard econometric methodology.

Cost-effectiveness analyses for the intervention will be undertaken based on findings from the costing studies and the trial described in this proposal. The cost-effectiveness analyses will be stochastic to account for the level of uncertainty uncovered in the trials. In such analysis, the

influence of the uncertainty is directly communicated, thus making the output more suitable for policy recommendations. Results from these economic evaluations will be integrated into the work of the Health Systems Research group.

5.0 Ethical considerations

This research raises sensitive ethical issues regarding human beings, human biological samples, and personal data but not regarding genetic information or animals. Applications for ethical clearance will be submitted to the medical ethics committees at all seven partner institutions.

All aspects of the study will be in agreement with the latest version of the Helsinki declaration and we will undertake the study in accordance to the guidelines of the "Consort" statement. We will also follow the UN convention on the Rights of the child and the Convention of the Council of Europe on Human Rights and Biomedicine signed in Oviedo on 4 April 1997. Participation is voluntarily; when enrolled, the caretakers are allowed to withdraw from the study without giving any reason and without the withdrawal having any impact on the subsequent services offered to the child. Written, informed consent will be sought; this will be read out loud to the caretakers of the eligible children in front of a witness who will sign for the respondent if necessary that oral permission has been given. A register over witnesses will be kept.

We will conduct a randomised trial in four African countries in which one arm will receive peer counselling to promote peer counselling, but both will receive an enhanced breastfeeding promotion package including mass media promotion of the concept of exclusive breastfeeding (EBF) and access to trained lactation management consultants connected to the local health care facility in the project sites. We have chosen peer counselling as the intervention to randomise (at individual level) because we believe it may have a large impact on EBF rates. Only a few studies have evaluated its impact; slightly over half of them have found an effect, but the others have not and thus we think it is ethically justifiable to randomise it. In particular, peer counselling has not yet been tested in Africa and the effectiveness of peer counsellors is likely to relate to cultural issues, which cannot be taken for granted. Given the enormous potential impact of EBF on infant health and mortality, if our trial is effective, we hope that health care systems will themselves implement it. To facilitate that process, we will also be measuring and documenting the costs, cost-effectiveness, and health system burden/implications of doing so.

Safety

Breastfeeding is safe and acceptable all over the world. The risk of HIV infection will be dealt with in the usual way as a part of voluntary counselling and testing for HIV. That is, the women will be offered HIV testing and if found HIV-positive they will make an informed choice after being provided with information on the advantages and disadvantages of each type of feeding. In

addition, we will be training and hiring lactation management consultants and linking them to the local health centres. They (and to a lesser extent the peer counsellors) will assist mothers to use the correct latching on, positioning and other factors to reduce the risk of breast damage with consequent increase in the risk of HIV transmission. Finally, the usual prophylaxis with nevirapine to the mother at the time of delivery and to the infant within two days after delivery will be offered. There is accordingly no risk of any serious adverse effects from participation in the PROMISE study.

We suggest to draw 5 ml of blood from a number of infants on two occasions during the first year of life. This is to clarify if exclusive breastfeeding up to 6 months of age may have a negative impact on the micronutrient status of the infant. In some instances the blood sample will beneficial for the infant itself. For instance, if the mother is HIV-positive and the family consents, these samples will be analysed for HIV and if the child has become HIV-positive, adequate care will be provided for the child. The blood will also be analysed for micronutrients and if a deficit is found, adequate supplementation will be given. In such instances, the ethical problem is small. But for many children, this blood sample will not benefit their own health directly and therefore it may be questioned whether it is justifiable to perform these blood samplings on infants. The problem is that the research question cannot be answered by studying adults. The alternative in this particular case is in fact not to attempt to answer the question.

WHO has called for an answer to this particular question, and we therefore consider the ethical dilemma as solvable. But of course this question will be scrutinised in all the medical ethics committees related to the involved institutions and we will of course follow their advice.

Prevention of spill over effects

There is anecdotal evidence that the promotion of exclusive breastfeeding (EBF), an usual activity in most African countries, has resulted in stigma when it is done as a part of VCT for HIV. We are going to great lengths to avoid this risk by briefly promoting EBF as social norm in the mass media at three points in time during the recruitment period.

6.0 Plan for using and disseminating knowledge

The acquired knowledge during the project will fall under the confidentiality agreement. Any information provided by a participant of the project to another participant of the project will be considered as confidential information and will not be transmitted in any format to a third party without the agreement of the participant who provided the information. The scientific outcome of the project is not only important for the partners but also for participating communities and the international scientific community. Dissemination plans for the spread of this knowledge include presentations at both scientific and policy-oriented conferences and meetings, and publication of

the scientific results of the project in peer-reviewed journals. We will also participate in educational efforts and transfer of knowledge to policy-makers and local authorities. A web site will be set up to present the results.

6.1 Management of knowledge and intellectual properties

The project participants expect that increased scientific knowledge will be the main outcome of this project. Management and dissemination of the knowledge will be applied to this outcome. Internal guidelines for authorship have already been agreed upon.

6.2 Publications

The following routes of dissemination will ensure the widest possible distribution:
Publication of major findings in a mainstream journal.
Publication of major findings in national journals by local collaborators.

7.0 Potential conflict of interest

None.

8.0 Project management structure

The project management structure consists of:

- Project Coordinator: Prof. Thorkild Tylleskär.
- The Steering Committee, consisting of the team leaders of each partner and the coordinator.

8.1 The coordinator

The overall project management will be the responsibility of the coordinator (CIH, Bergen). The overall coordinator will be responsible for:

- management of the project and reporting to the Steering Committee and executing its decisions. This consists of verification and adjustments of the project progress, discussion on the scientific results
- organisation and chairing of the quarterly call conferences and the annual meetings with the Steering committee
- ultimate responsibility for the financial and administrative reporting to the European Commission
- managing the financial administration of the overall project expenses including reporting the overall budgetary situation of the project to the European Commission every year, based on the cost declarations of the individual partners, as stated in the contract

- monitoring the cost declarations of the individual partners every three months and in case of misunderstandings or errors undertake immediate action
- answer questions of the individual partners and the European Commission in the area of administration and financial aspects of the projects
- the overall respect of the ethical and gender issues within the project
- avoiding conflicts. The project coordinator will try to solve conflicts or to reach a compromise with the Steering committee

8.2 Steering Committee

The Steering committee has the responsibility on the strategic issues of the project and therefore will consist of all partners. Each partner will be represented by its coordinator/team leader as follows:

1. Prof Thorkild Tylleskär, (CIH), University of Bergen, Norway
2. Prof Philippe Van de Perre, University of Montpellier (UMR145), France
3. Researcher Eva-Charlotte Ekström, PhD (IMCH), Univ. of Uppsala, Sweden
4. Dr Nicolas Meda, Centre Muraz, Burkina Faso
5. Prof. James Tumwine, Makerere University. (MAK), Uganda
6. Dr Chipepo Kankasa, University of Zambia, School of Med. (UNZA/SOM)
7. Dr Debra Jackson, University of Western Cape (UWC), Cape Town, South Africa

The role of the Steering committee is to ensure that the research activities are executed in a consistent and standardised way. The Steering committee will meet annually to discuss the activities, scientific and technical aspects of the study.

The Steering committee will be responsible for:

- monitoring the progress of the project, including progress in workpackage timing, verification of the deliverables, and verification of achievement of the milestones
- overall responsibility for the project reporting (progress reports, midterm assessment and end report) to the European Commission
- organisation and transmission of technical and scientific project information to and between the WP leaders
- deciding on major changes in the project plans
- handling any disputes among partners

9. Logical time framework

Schedule of workpackages

Year	1						2						3						4					
Months	1<br>2	3<br>4	5<br>6	7<br>8	9<br>10	11<br>12	13<br>14	15<br>16	17<br>18	19<br>20	21<br>22	23<br>24	25<br>26	27<br>28	29<br>30	31<br>32	33<br>34	35<br>36	37<br>38	39<br>40	41<br>42	43<br>44	45<br>46	47<br>48
Workpackage description																								
WP1 Formative research on infant feeding practices																								
WP2 Lactation management																								
WP3 Training of peer counsellors for EBF																								
WP3.9 Standard operating procedures for the trial																								
WP4 Randomised EBF trial Burkina Faso																								
WP5 Randomised EBF trial Uganda																								
WP6 Randomised EBF trial Zambia																								
WP7 Randomised EBF trial South Africa																								
WP8 Micronutrient analysis																								
WP9 Cost-effectiveness analysis																								
WP10 Health systems analysis																								
WP11 Data collection, validation, management, analysis																								
WP12. Dissemination																								
WP13.1 Project management																								
WP13.2 Project meetings																								

10.0 References

1. Jones G, Steketee RW, Black RE, Bhutta ZA, Morris SS. How many child deaths can we prevent this year? *Lancet*. 2003; **362**: 65-71.
2. Black RE, Morris SS, Bryce J. Where and why are 10 million children dying every year? *Lancet*. 2003; **361**: 2226-34.
3. Almroth SG. Water requirements of breast-fed infants in a hot climate. *American Journal of Clinical Nutrition*. 1978; **31**: 1154-57.
4. WHO Division of Child Health and Development. Indicators for assessing breastfeeding practices. Geneva: WHO; 1991. Report No.: WHO/CDD/SER/91.14.
5. Labbok M, Krasovec K. Towards consistency in breastfeeding definitions. *Studies in Family Planning*. 1990; **21**: 226-30.
6. WHO. The optimal duration of exclusive breastfeeding: a systematic review; 2002.
7. Greiner T. History of breastfeeding. *Nursing Mothers' Association of Australia Newsletter*. 1998; **34**: 4-5.
8. Coutsoudis A, Goga AE, Rollins N, Coovadia HM. Free formula milk for infants of HIV-infected women: blessing or curse? *Health Policy Plan*. 2002; **17**: 154-60.
9. Kuhn L, Stein Z. Infant survival, HIV infection, and feeding alternatives in less-developed countries. *Am J Public Health*. 1997; **87**: 926-31.
10. Coutsoudis A, Pillay K, Kuhn L, Spooner E, Tsai WY, Coovadia HM. Method of feeding and transmission of HIV-1 from mothers to children by 15 months of age: prospective cohort study from Durban, South Africa. *Aids*. 2001; **15**: 379-87.
11. Latham MC, Kisanga P. Current status of protection, support and promotion of breastfeeding in four African countries.: UNICEF ESARO; 2001.
12. Fawzi WW, Msamanga GI, Hunter D, Renjifo B, Antelman G, Bang H, et al. Randomized trial of vitamin supplements in relation to transmission of HIV-1 through breastfeeding and early child mortality. *Aids*. 2002; **16**: 1935-44.
13. John GC, Nduati RW, Mbori-Ngacha DA, Richardson BA, Panteleeff D, Mwatha A, et al. Correlates of mother-to-child human immunodeficiency virus type 1 (HIV-1) transmission: association with maternal plasma HIV-1 RNA load, genital HIV-1 DNA shedding, and breast infections. *Journal of Infectious Disease*. 2001; **183**: 206-12.
14. Pillay K, Coutsoudis A, York D, Kuhn L, Coovadia HM. Cell-free virus in breast milk of HIV-1-seropositive women. *J Acquir Immune Defic Syndr*. 2000; **24**: 330-6.
15. Semba RD, Kumwenda N, Taha TE, Hoover DR, Quinn TC, Lan Y, et al. Mastitis and immunological factors in breast milk of human immunodeficiency virus-infected women. *J Hum Lact*. 1999; **15**: 301-6.
16. Willumsen JF, Filteau SM, Coutsoudis A, Newell ML, Rollins NC, Coovadia HM, et al. Breastmilk RNA viral load in HIV-infected South African women: effects of subclinical mastitis and infant feeding. *AIDS*. 2003; **17**: 407-14.
17. Nduati R, John G, Mbori-Ngacha D, Richardson B, Overbaugh J, Mwatha A, et al. Effect of breastfeeding and formula feeding on transmission of HIV-1: a randomized clinical trial. *JAMA*. 2000; **283**: 1167-74.
18. Miotti PG, Taha TE, Kumwenda NI, Broadhead R, Mtimavalye LA, Van der Hoeven L, et al. HIV transmission through breastfeeding: a study in Malawi. *JAMA*. 1999; **282**: 744-9.
19. Embree JE, Njenga S, Datta P, Nagelkerke NJ, Ndinya-Achola JO, Mohammed Z, et al. Risk factors for postnatal mother-child transmission of HIV-1. *AIDS*. 2000; **14**: 2535-41.
20. Piwoz EG, Ross J. HIV and infant feeding: Knowledge, gaps, and challenges for the future. In: Greiner T, de Wagt A, Labbok M, editors. HIV & Infant Feeding, Report of a WABA-UNICEF Colloquium. Penang: World Alliance for Breastfeeding Action and UNICEF; 2003. p. 29-43.
21. Kramer MS, Kakuma R. The Optimal Duration of Exclusive Breastfeeding, a Systematic Review. Geneva: WHO; 2002. Report No.: WHO/NHD/01.08.
22. WHO. Infant and young child nutrition. 54th World Health Assembly; 2001 18 May 2001; Geneva; 2001.
23. Dewey KG, Cohen RJ, Brown KH, Rivera LL. Effects of exclusive breastfeeding for four versus six months on maternal nutritional status and infant motor development: results of two randomized trials in Honduras. *J Nutr*. 2001; **131**: 262-7.

24. WHO. Report of the Expert Consultation on the Optimal Duration of Exclusive Breastfeeding. Geneva: WHO; 2001 28-30 March 2001. Report No.: WHO/NHD/01.09.
25. Butte NF, Lopez-Alarcon MG, Garza C. Nutrient Adequacy of Exclusive Breastfeeding for the Term Infant During the First Six Months of Life. Geneva: WHO; 2002.
26. Kramer MS, Guo T, Platt RW, Sevkovskaya Z, Dzikovich I, Collet JP, et al. Infant growth and health outcomes associated with 3 compared with 6 mo of exclusive breastfeeding. *Am J Clin Nutr*. 2003; **78**: 291-5.
27. Hutton G, Wyss K, N'Diekhor Y. Prioritization of prevention activities to combat the spread of HIV/AIDS in resource constrained settings: a cost-effectiveness analysis from Chad, Central Africa. *Int J Health Plann Manage*. 2003; **18**: 117-36.
28. Grummer-Strawn LM, Rice SP, Dugas K, Clark LD, Benton-Davis S. An evaluation of breastfeeding promotion through peer counseling in Mississippi WIC clinics. *Matern Child Health J*. 1997; **1**: 35-42.
29. Schafer E, Vogel MK, Viegas S, Hausafus C. Volunteer peer counselors increase breastfeeding duration among rural low-income women. *Birth*. 1998; **25**: 101-6.
30. Gross SM, Caulfield LE, Bentley ME, Bronner Y, Kessler L, Jensen J, et al. Counseling and motivational videotapes increase duration of breast-feeding in African-American WIC participants who initiate breast-feeding. *J Am Diet Assoc*. 1998; **98**: 143-8.
31. Shaw E, Kaczorowski J. The effect of a peer counseling program on breastfeeding initiation and longevity in a low-income rural population. *J Hum Lact*. 1999; **15**: 19-25.
32. Martens PJ. Increasing breastfeeding initiation and duration at a community level: an evaluation of Sagkeeng First Nation's community health nurse and peer counselor programs. *J Hum Lact*. 2002; **18**: 236-46.
33. Kistin N, Abramson R, Dublin P. Effect of peer counselors on breastfeeding initiation, exclusivity, and duration among low-income urban women. *J Hum Lact*. 1994; **10**: 11-5.
34. Haider R, Ashworth A, Kabir I, Huttly SR. Effect of community-based peer counsellors on exclusive breastfeeding practices in Dhaka, Bangladesh: a randomised controlled trial. *Lancet*. 2000; **356**: 1643-7.
35. Morrow AL, Guerrero ML, Shults J, Calva JJ, Lutter C, Bravo J, et al. Efficacy of home-based peer counselling to promote exclusive breastfeeding: a randomised controlled trial. *Lancet*. 1999; **353**: 1226-31.
36. Caulfield LE, Gross SM, Bentley ME, Bronner Y, Kessler L, Jensen J, et al. WIC-based interventions to promote breastfeeding among African-American Women in Baltimore: effects on breastfeeding initiation and continuation. *Journal of Human Lactation*. 1998; **14**: 15-22.
37. McInnes RJ, Love JG, Stone DH. Evaluation of a community-based intervention to increase breastfeeding prevalence. *J Public Health Med*. 2000; **22**: 138-45.
38. Green CP. Interventions to improve breastfeeding behaviors: detailed summaries of 51 studies. Washington, DC: LINKAGES; 1999.
39. Nash S. Does exclusive breast-feeding reduce the risk of coeliac disease in children? *Br J Community Nurs*. 2003; **8**: 127-32.
40. Hilbig A, Kersting M, Sichert-Hellert W. Measured consumption of tap water in German infants and young children as background for potential health risk assessments: data of the DONALD Study. *Food Addit Contam*. 2002; **19**: 829-36.
41. Rao MR, Hediger ML, Levine RJ, Naficy AB, Vik T. Effect of breastfeeding on cognitive development of infants born small for gestational age. *Acta Paediatrica*. 2002; **91**: 267-74.
42. Kramer MS, Chalmers B, Hodnett ED, Sevkovskaya Z, Dzikovich I, Shapiro S, et al. PROBIT Study Group (Promotion of Breastfeeding Intervention Trial). *JAMA*. 2001; **285**: 413-20.
43. Silfverdal SA, Bodin L, Hugosson S, Garpenholt O, Werner B, Esbjorner E, et al. Protective effect of breastfeeding on invasive Haemophilus influenzae infection: a case-control study in Swedish preschool children. *Int J Epidemiol*. 1997; **26**: 443-50.
44. Martines J. HIV and infant feeding--a Framework for Priority Actions. In: Greiner T, al. e, editors. HIV & Infant Feeding; a report of a WABA-UNICEF Colloquium. Penang, Malaysia: WABA and UNICEF; 2003. p. 21-4.

11.0 Appendices
