# Supplementary material for: Effect of Breastfeeding Promotion on Early Childhood Caries and Breastfeeding Duration among 5 Year Old Children in Eastern Uganda: A Cluster Randomized Trial
Source: PLoS One. 2015 May 4;10(5):e0125352. doi: 10.1371/journal.pone.0125352 (PMC4418833; doi:10.1371/journal.pone.0125352)
Supplement: S1 Table — (PDF) [file pone.0125352.s005.pdf]

Table S1A showing zero inflated model with Vuong’s test (n=417)

	Beta coefficient	95% confidence intervals
**First step (binomial count variable)**		
Intervention	0.015	(-0.37− 0.34)
Control	0	0
**Second step (non-zero ECC outcome)**		
Intervention	0.17	(-0.41− 0.75)
Control	0	0

ECC- Early childhood caries
Zinb- zero inflated negative binomial regression
Vuong’s test of zinb vs. standard negative binomial: z = 2.09, Pr>z = 0.0184
Vuong's test does not take clustering into account
$p < 0.05$

Table S1B showing zero inflated model with incidence rate ratios and odds ratio

**First step (binomial count variable)**		
	**IRR**	**95% confidence intervals**
Intervention	0.99	(0.70−1.39)
Control	1	
**Second step (non-zero ECC outcome)**		
	**OR**	**95% confidence intervals**
Intervention	1.19	(0.71−1.99)
Control	1	

Incidence rate ratio - IRR, Odds ratio- OR
